# The Immune/Inflammatory Underpinnings of Neurodevelopmental Disorders and Pediatric Acute-Onset Neuropsychiatric Syndrome: A Scoping Review

**DOI:** 10.3390/ijms26167767

**Published:** 2025-08-11

**Authors:** Antonella Gagliano, Francesca Cucinotta, Ivana Giunta, Irene Di Modica, Carmela De Domenico, Carola Costanza, Eva Germanò, Jennifer Frankovich

**Affiliations:** 1 Department of Medicine and Surgery, “Kore” University of Enna, 94100 Enna, Italy; antonellagagliano.npi@gmail.com; 2 Oasi Research Institute-IRCCS, 94018 Troina, Italy; carola.costanza@unipa.it; 3 IRCCS Centro Neurolesi Bonino Pulejo, S.S. 113 Via Palermo, C. da Casazza, 98124 Messina, Italy; carmela.dedomenico@irccsme.it; 4 Child and Adolescent Neuropsychiatry Unit, Department of Human and Evolutionary Pathology “Gaetano Barresi”, University of Messina, 98122 Messina, Italy; ivanagiunta7@gmail.com (I.G.); d.irene2795@gmail.com (I.D.M.); eva.germano@unime.it (E.G.); 5 Department of Allergy, Immunology, Rheumatology, Division of Pediatrics, Stanford University School of Medicine, Palo Alto, CA 94305, USA; jfranko@stanford.edu; 6 Immune Behavioral Health Clinic and Research Program, Stanford University Children’s Hospital, Palo Alto, CA 94304, USA

**Keywords:** neuroinflammation, neuroimmunology, neurodevelopmental disorders, PANS, pathogenetic pathways

## Abstract

Among the shared pathogenetic mechanisms leading to neurodevelopmental disorders (NDDs), a dysregulated inflammatory response has been described as a convergent pathway in NDDs. This scoping review was registered in the OSF database. It was conducted in accordance with the PRISMA Extension for Scoping Reviews, utilizing a comprehensive literature search of major academic databases, including PubMed and Web of Science. The search was performed until 1 March 2025, using a combination of predefined search terms and Boolean operators (AND, OR) to ensure a comprehensive identification of relevant studies. A comprehensive summary of the evidence on immunological and neuroinflammatory pathways underlying the NDDs is shown. This review also reports evidence on early-onset presentation of schizophrenia spectrum and obsessive–compulsive disorder since clinical researchers are beginning to consider these conditions neurodevelopmental disorders. Furthermore, this review outlines the recently described clinical entity, PANS (Pediatric Acute-Onset Neuropsychiatric Syndrome), and its clinical and pathogenetic contact points with NDDs, delineating a spectrum of disorders that share common pathogenetic pathways. This scoping review improves the awareness of immune/neuroinflammatory correlates supporting NDDs. Furthermore, it suggests adopting a transnosographic approach to neuropsychiatric disorders, including PANS as a syndromic construct that overlaps with NDDs.

## 1. Introduction

“Neurodevelopmental disorders” (NDDs) are presented in the DSM-5 [1] as an overarching disorder category including intellectual disability (ID), communication disorders (CDs), autism spectrum disorder (ASD), attention deficit/hyperactivity disorder (ADHD), specific learning disorders (SLDs), and motor disorders, such as Tourette’s disorder (TD). NDDs have a multifactorial etiology [2]. These disorders are categorized into discrete disease entities based solely on clinical presentation [1] because specific biomarkers to diagnose or differentiate between them are not available. On the contrary, a broad range of common neurobiological underpinnings have been implicated in the expression of genes involved in both prenatal and postnatal mechanisms, which influence synaptic activity and plasticity [3]. Among the shared pathogenetic routes, a dysregulated inflammatory response has been described as a convergent pathway in NDDs [4,5,6].

The development of the central nervous system (CNS) is a dynamic, multi-componential process that extends from conception through adolescence and is driven by a complex interplay of genetic, epigenetic, neurobiological, and environmental factors [7]. In the fetal brain, 86 billion neurons must be generated in a highly coordinated and precise manner to form complex functional networks. Even small changes in this complex process can lead to structural and functional weaknesses that have lasting and significant effects on brain function [8]. Emerging data point to problematic interactions between the immune system and the CNS as a factor that may disrupt normal neurodevelopment and resilience. The immune system not only protects the body from infections and pathogens but also influences how the brain develops and maintains its function over time [4]. Proper functioning of the immune system requires responses from both innate and adaptive immunity, with the interaction of a broad range of mechanisms in dynamic and harmonized pathways. This complex nature of the immune system leads to a high liability for dysregulations and, consequently, to a plethora of conditions, including psychiatric disorders [9].

A new branch of science, known as “immunoneuropsychiatry”, now offers integrated and innovative views on immune abnormalities in neuropsychiatric disorders [10], highlighting the crucial role of the immune system in CNS development and function. As a result of this new integrated perspective, there is increasing recognition of inflammation/immune-mediated neuropsychiatric disorders [11,12]. The key evidence supporting the immune system’s involvement in NDDs comes from studies examining the links between infections and neuropsychological and psychiatric symptoms [13,14]. Many scientific advances indicate that normal cognitive and neurological functioning depends on a delicate balance, which is influenced by activities of the immune system from the earliest stages of CNS development. In particular, the centrality of the first 1000 days (from conception to 2 years of age) has been highlighted, establishing that this sensitive period of a child’s life is crucial for physical, neural, cognitive, and social–emotional development [15]. For example, maternal exposure to pathogens during pregnancy has long been considered a strong risk factor for NDDs and schizophrenia (SCZ) in offspring [16,17,18]. Well-recognized pathophysiological pathways in animal models, together with emerging human evidence, account for an association between immune/inflammatory perturbations in utero and offspring neurodevelopmental disorders [19], in particular ASD [20] and SCZ [21]. The formulation of this hypothesis, known as “Maternal Immune Activation” (MIA) [22], arises from both animal models and extensive epidemiological research, which suggests a critical role for cytokine-associated inflammatory events during pregnancy. Consistent with this hypothesis, any maternal condition associated with immune activation (e.g., autoimmune diseases, infections, allergies) may impact fetal CNS development [19,23]. MIA may affect vulnerable offspring through various mechanisms, including epigenetic changes, which can manifest at different time points in the offspring’s development and life course, as well as in subsequent generations [24].

An example of a postnatal immune–brain epoch that involves the adaptive immune system is autoimmune encephalitis (AE), a disease associated with a range of symptoms, including psychiatric symptoms [25,26,27]. AE comprises a set of disorders in which the host’s immune system targets self-antigens expressed in the CNS via autoantibodies directed against neuronal proteins, ion channels, and receptors [25,26,27,28,29]. AE clinical features are determined by the target of the autoantibodies and subsequent brain areas and circuits involved, and where the brain barriers are disrupted (as this is a requirement for systemic autoantibodies to access the brain) [30,31]. The CNS has historically been regarded as an immune-privileged environment due to the protection provided by specialized physical barriers (the blood–brain barrier and the blood–lymphatic barrier), which, in normal circumstances, stop circulating immune products from entering the CNS. However, various conditions, such as infection, hypoxia, and trauma, can disrupt these barriers, allowing infections, toxins, and immune cells, as well as their products, to enter and alter CNS circuits and also activate the resident CNS immune system [10,32,33,34]. An illustrative example of this process is a mouse model in which repeated Group A Streptococcus (GAS) infections induce the migration of Th17 lymphocytes from the nasal lymphoid tissue (equivalent structure to the human tonsils) into the brain through the olfactory bulb, which leads to inflammation and injury/disruption of the blood–brain barrier, microglial activation, and a loss of synaptic proteins [35].

Microglia are the resident immune cells of the brain, playing a crucial role in both neurodevelopment and responding to pathogens and injury by triggering an inflammatory response. Microglia contribute to brain development through their involvement in neuronal proliferation and migration, astrogenesis and astrocyte development, myelinogenesis and synaptogenesis, and neural circuit plasticity [36,37,38]. During neurodevelopment, infections, toxins (e.g., alcohol and illicit drugs), and genetic risk factors (e.g., mutations in MECP2 and UHRF1) can cause epigenetic dysregulation in microglia, including the expression of microRNAs, which leads to neuroinflammation and thereby affects neurodevelopment [39]. The inflammasome (which is a multi-protein cytosolic complex contained within many cells, including microglia), via complex mechanisms, prompts the release of proinflammatory cytokines and contributes to neuroinflammation, which impacts neurologic functioning and alters behavior [40,41,42,43]. Microglia exhibit a variety of phenotypes, including the proinflammatory (M1) and anti-inflammatory (M2) phenotypes. The M1 phenotype produces several proinflammatory molecules (e.g., interleukin-1β, -6, and -12, TNF α, and reactive oxygen species); the M2 phenotype produces anti-inflammatory molecules and neuroprotective factors (e.g., insulin-like growth factor 1 and brain-derived neurotrophic factor, interleukin-4, -10, and -13) [44,45,46,47].

Both the adaptive immune response (antigen-specific reactivity and antibody production) and the innate immune response (microglial activation and cytokine production) are thought to be involved in a spectrum of CNS disorders, and this review will outline the involvement of these arms in NDDs. Most of these NDDs have symptoms which may wax and wane (ADHD, OCD, Tourette’s, etc.) or relapse and remit (PANS). This fluctuating course is true with most inflammatory conditions (asthma, eczema, psoriasis, arthritis, etc.). We acknowledge that the ramping-up phase of a condition (flare onset to peak symptoms) will reflect different immune events/interactions/profiles compared to the recovery or improving phase (peak symptoms to baseline). Additionally, there are likely differences based on subgroups of each of the different NDDs (chronic–static cases, primary progressive, single episode, frequent relapses, secondary chronic, etc.). Additionally, the influence of season (fall/winter/early spring carries a higher infection burden compared to summer) on study protocols will also influence study findings. We mention this upfront since, like inflammatory diseases, immunological findings in NDDs and PANS will vary based on study design (phase of disease studied, subgroups analyzed, collection protocols with regards to seasons, etc.).

### Aim of Review

This scoping review intends to provide a summary of the evidence on immunological and neuroinflammatory pathways underlying NDDs. It also reports evidence on the early-onset presentation of schizophrenia spectrum and other psychotic disorders (SS/PDs) and on obsessive–compulsive disorder (OCD), since several researchers have concluded that these conditions should be considered neurodevelopmental disorders [48,49]. Furthermore, this review outlines the recently described clinical entity, PANS (Pediatric Acute-Onset Neuropsychiatric Syndrome), and its clinical and pathogenetic connections with NDDs [50], with the purpose of delineating a spectrum of disorders that share common pathogenetic pathways.

## 2. Materials and Methods

### 2.1. Data Source and Search Strategy

This scoping review was registered in the OSF database (DOI 10.17605/OSF.IO/79FHD). The study was conducted in accordance with the PRISMA Extension for Scoping Reviews (PRISMA-ScR) [51], as outlined in the PRISMA-ScR checklist (Supplementary Table S1). A comprehensive literature search was conducted across major academic databases, including PubMed and Web of Science. The search was performed until 1 March 2025, using a combination of predefined search terms and Boolean operators (AND, OR) to ensure a comprehensive identification of relevant studies. The search strategy utilized both keywords and Medical Subject Heading (MeSH) terms to enhance specificity and sensitivity. Two principal search strings, performed in both databases, were evaluated separately: (1) (neuroinflammation) AND (“autism spectrum disorder” OR “Attention Deficit Hyperactivity Disorder” OR “Tourette Disorder” OR “intellectual disability” OR “pediatric Acute-Onset Neuropsychiatric Syndrome”); (2) (immune dysfunction) AND (“autism spectrum disorder” OR “Attention Deficit Hyperactivity Disorder” OR “Tourette Disorder” OR “intellectual disability” OR “pediatric Acute-Onset Neuropsychiatric Syndrome”).

Then, to increase the number of studies to screen, a third broader search was conducted after evaluating the initial results of the two more specific queries, aiming to identify potentially relevant studies that may have used less specific terminology. It was performed using the following key terms: neuroinflammation and neurodevelopmental disorders. In addition, reference lists of included studies or reviews were searched to ensure that a comprehensive list of relevant articles was considered for inclusion. Inclusion criteria were based on (a) children and adolescents (0–18 years); (b) randomized and non-randomized clinical trials, observational studies, systematic reviews, and meta-analyses published in indexed journals with peer-review processes; (c) language, specifically Italian, English, Spanish, or studies in another language for which translation was available; (d) availability of the full-text article. Studies published in English, Italian, and Spanish were included, as these are the languages in which the authors are proficient, ensuring accurate interpretation of the meaning without the need for translation, which could have introduced interpretive bias.

Exclusion criteria included (a) adult samples; (b) textbooks, editorials, letters to the editor, and single-case reports (n < 2); (c) languages other than Italian, English, and Spanish; (d) unavailability of the full-text article; (e) contents not connected to the topic of our review.

### 2.2. Selection Procedures

Study selection was conducted by two blinded authors (CDD, IDM). After removing duplicates, references were initially screened by title and abstract. Full-text articles were then evaluated for eligibility based on title, abstract, full-text content, and topic specificity. In cases of disagreement, a third author (FC) was consulted to reach a consensus. When overlapping studies were identified, the most comprehensive study was selected for inclusion.

### 2.3. Data Extraction and Evaluation

Two authors independently searched and extracted data (CDD, IDM) to describe and assess study characteristics. The coding process included interim and final concordance checks to ensure consistency and reliability. All variables were coded as categorical and clearly defined by one author (FC). Any discrepancies identified during interim checks were discussed and resolved by consensus between authors to ensure alignment before proceeding. In the final stage, a thorough review confirmed complete agreement on coding and article synthesis. Data extraction clusters included (1) neuroinflammatory, etiopathological, and pathophysiological mechanisms; (2) clinical manifestations; (3) a summary of primary treatment options and potential therapeutic strategies.

## 3. Results

**Figure 1:**
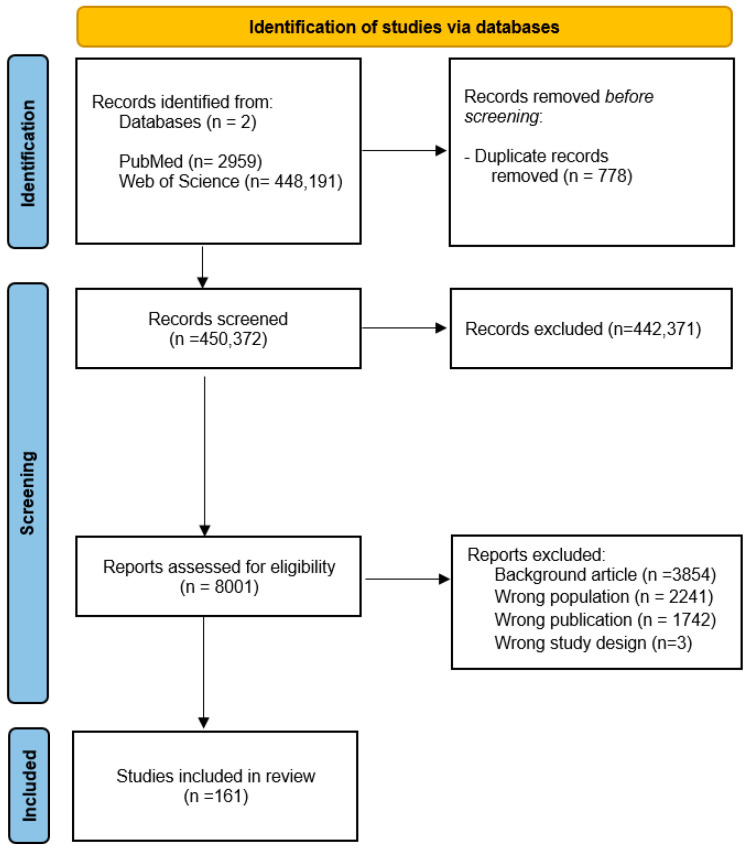
The PRISMA flow diagram indicating the process of selecting sources of evidence included in the scoping review. The numbers reported in the Record Identified reflect the cumulative results retrieved from three separate search strategies across two databases (PubMed and Web of Science) prior to the removal of duplicates.

The literature search yielded a total of 451,150 results. The entire selection process is reported in Figure 1. After removing 778 duplicates, we excluded 442,371 following the screening procedure; we then identified 8001 which were assessed for eligibility. Finally, a total of 161 studies were included: 6 randomized controlled trials (RCTs), 61 observational studies (cohort, case–control, cross-sectional), 28 descriptive studies, 57 literature reviews, 3 theoretical or conceptual papers, and 6 technical reports, theses, conference proceedings, and book chapters. The studies varied considerably regarding diagnostic criteria, age of onset, and methodological quality. For more details on the characteristics of the included studies, see Supplementary Table S2.

### 3.1. Evidence of Immune Dysregulation and Neuroinflammation in Neurodevelopmental Disorders

The etiopathogenetic correlates and susceptibility mechanisms previously described are relatively frequent and are not specific to a single disorder. Indeed, several neurodevelopmental disorders show evidence of association with immune disorders, maternal exposure to infections and toxic agents, or maternal immune activation during pregnancy.

### 3.2. Attention Deficit Hyperactivity Disorder

A potential role for neuroinflammation in ADHD has been primarily postulated based on the frequent association between ADHD and various immune disorders, such as eczema, asthma, rheumatoid arthritis, type 1 diabetes, psoriasis, and inflammatory bowel disease [52,53,54,55,56,57]. For example, in an extensive prevalence study on a cohort of 2500 individuals, there was a significant association between ADHD, psoriasis, and inflammatory bowel disease. The association persisted even after adjusting for smoking and body mass index, which were identified as potential confounding factors.

A co-aggregation of ADHD and autoimmune diseases has also been described among family members of ADHD probands, suggesting the presence of shared genetic risk factors between these two conditions [53,58,59,60]. Several immune diseases, such as multiple sclerosis, rheumatoid arthritis, type 1 diabetes, asthma, and hypothyroidism, appear to be more frequent among mothers of offspring with ADHD compared with mothers of neurotypical individuals [61]. Evidence from genetic studies suggests that polymorphisms in genes linked to inflammatory pathways contribute to the development of ADHD [62]. Genome-wide association studies (GWASs) confirmed the associations between ADHD and genes that regulate the inflammatory cascade [63,64], supporting the hypothesis of genetic vulnerability to immune activation. However, a population-based cohort study [65] showed a stronger association in mothers than in fathers between some familial autoimmune/allergic diseases and ADHD and ASD. This evidence supports a role for MIA in ADHD vulnerability. Mouse models support the theory that MIA alters brain development, including a reduction in cortical gray matter in brain areas implicated in ADHD (e.g., prefrontal cortex) [66]. In particular, MIA appears related to the expression of Sonic Hedgehog Protein (SHH), one of the ligands of the ‘hedgehog signaling pathway,’ a fundamental pathway in the regulation of organogenesis, including CNS development. SHH plays a critical role in the maturation of dopaminergic neurons and the development of the dopaminergic system [67]. A strong epidemiological validation of MIA mouse models is supported by a large Danish population-based mother–child cohort of 700 mother–child pairs, followed prospectively from pregnancy week 24 to 10 years of age [68]. Higher maternal high-sensitivity C-reactive protein levels in pregnancy were significantly associated with offspring ADHD diagnosis by age 10 (OR 1.40, 95%CI, *p* = 0.001).

Furthermore, higher maternal C-reactive protein levels in pregnancy predicted ADHD symptom load in the whole cohort, demonstrating a robust association of prenatal maternal inflammation with the ADHD spectrum. Finally, in individuals with ADHD, higher levels of antibodies against the basal ganglia and/or the dopamine transporter were detected [57,60], along with an imbalance between pro- and anti-inflammatory cytokines, which correlates with symptom severity [69,70,71]. A comprehensive review found that inflammatory disorders, altered immune response, genetic and environmental associations, and polymorphisms in inflammatory-related genes may increase neuroinflammation in the ADHD population [72]. At the same time, the review highlighted the role of the imbalance between oxidants and antioxidants as a factor potentially leading to oxidative damage. In summary, ADHD is a disorder with a highly heterogeneous genetic and clinical presentation. Therefore, the ADHD population exhibits both genetic and environmental immune-related risk factors [73], encompassing polymorphisms in inflammatory-related genes, maternal immune activation with an impact on offspring brain development, associations with autoimmune and inflammatory diseases, altered immune responses to infectious agents, and stress.

### 3.3. Autism Spectrum Disorder

Beyond the two main symptoms (deficits in communication/social interaction and restricted/repetitive interests/activities), ASD is often associated with additional symptoms, including sensory dysregulation, emotion dysregulation, motor impairments, cognitive/learning impairment, and ADHD symptoms [1]. The ASD population also suffers from psychiatric conditions (anxiety, depression, obsessive-compulsive disorder, schizophrenia, bipolar disorder) more frequently than the neurotypical population [74]. Finally, people with ASD may have a range of neurological and physical conditions, such as tuberous sclerosis, chromosome abnormalities, epilepsy, sleep disorders, feeding issues (e.g., food selectivity), and gastrointestinal problems [75,76,77,78,79,80,81]. A broad spectrum of symptoms can only be supported by a multifactorial etiopathogenesis, in which genetic, environmental, and inflammatory factors contribute.

A wide range of research data converge on the role of the immune system in ASD [82,83,84,85]. Evidence from longitudinal, population-based birth cohorts highlights a high risk of autoimmune disorders in patients with ASD [86]. Several immune-related conditions, including allergies and autoimmune diseases (especially psoriasis), seem to be more frequent in children with ASD compared to neurotypical individuals [87]. Secondarily, infective and inflammatory conditions during pregnancy are associated with an increased risk of ASD in offspring. For example, an extensive Swedish population study confirmed that maternal infections during pregnancy increase the risk of autism and depression in offspring [88]. Beyond infection, several MIA conditions during pregnancy seem to enhance ASD risk in offspring. In a case–control study performed in California [89], mothers with asthma (OR = 1.62) or obesity (OR = 1.51) were more likely to have infants with ASD. The risk further increased among female children if mothers had both asthma and extreme obesity (OR = 16.9). Among the offspring of mothers with elevated levels of autoantibodies involved in autoimmune thyroiditis (e.g., Thyroid Peroxidase Antibody—TPO-Ab) during pregnancy, the odds of autism increased by 80% (OR = 1.78) [90].

An Australian population-based cohort study [91] on the potential synergistic effects between maternal autoimmune disease and early childhood infections on ASD risk in offspring revealed no evidence of an additive interaction between the two factors. At the same time, both maternal autoimmune disease (OR 1.25) and childhood infection before age 2 years (OR 1.38) resulted in increased odds of offspring ASD. Reliably, several studies have found that elevated levels of maternal proinflammatory markers increase the risk of ASD and schizophrenia. In particular, maternal cytokines and chemokines during gestation are related to ASD and intellectual disability [92,93]. Strong evidence suggests that elevated maternal levels of IL-6 are associated with changes in the connectivity of the amygdala and fronto-limbic white matter in newborns, which in turn are linked to working memory and cognitive impairment later in life [94,95]. In parallel, cytokine and chemokine levels in newborns’ serum have been linked to an elevated risk of developing ASD [96,97,98].

According to the MIA hypothesis, chronic fetal neuroglial activation may lead to harmful effects on neurogenesis, migration, synapse formation, and pruning, crucial for the occurrence of ASD [20]. The MIA inflammatory status also results in oxidative stress in the placenta and fetal brain [99]. These factors interfere with neurodevelopment and represent the epigenetic “priming” of fetal microglia and postnatal immune–brain crosstalk [20,100,101]. Individuals exposed to MIA maintain a long-lasting epigenetic memory in fetal microglia and immune cells [102], with an impact on CNS development, particularly during critical periods of life, such as adolescence [23]. In mouse models, elevated blood cytokine levels, microglial activation, increased expression of proinflammatory cytokines, and increased oxidative stress in the cerebral cortex have been detected in adolescent MIA offspring, along with pathological changes in synaptic ultrastructure, deficits in presynaptic proteins, and downregulation of postsynaptic scaffolding proteins [101]. In recent decades, considerable research has focused on microglial activation and astrocyte changes underlying neuroinflammation and synaptic susceptibility [103,104], as well as brain alterations in ASD [105,106]. Notably, 20 years ago, clear evidence of active neuroinflammatory processes in the cerebral cortex, white matter, and cerebellum, along with the activation of microglia and astroglia, and a proinflammatory profile of cerebrospinal fluid (CSF) cytokines was found in postmortem ASD brain tissues and CSF from living individuals with ASD [107]. More recent studies have confirmed these findings, analyzing the composition of CSF among ASD subjects and finding elevated concentrations of four inflammatory cytokines (TNF-α, IL-4, IL-21, and BAFF) compared to controls [108].

In addition to the aforementioned alterations in CNS immunity, differences in peripheral immunity, such as a reduction in regulatory B cells and T cells, have been demonstrated in individuals with ASD compared to neurotypical subjects [109]. According to the authors, the immunological imbalance between regulatory B and T cells may play a role in the development of immune deficiencies and autoimmune diseases, which in turn impact the brain. Nevertheless, among a broad population of 25,088 individuals affected by primary immunodeficiency disorders (PIDs), ASD had the lowest prevalence (0.9%), compared to all mental health disorders, such as major depressive disorder (29%) and self-harm behaviors (9.3%) [110]. Overall, individuals diagnosed with ASD appear to exhibit immune dysregulation, as demonstrated by altered levels of autoantibodies and immunoglobulins, as well as elevated peripheral cytokines and chemokines [111].

### 3.4. Tics and Tourette’s Disorder

Tics and Tourette syndrome (TS) refer to a group of neurodevelopmental disorders that typically commence during childhood and adolescence and have a chronic or fluctuating course [1]. The prevalence is around 1% between 5 and 18 years of age, and the prevalence is higher in children than in adults, with the highest occurrence between 7 and 11 years of age [112,113]. The etiopathogenesis is attributable to an interaction between genetic and epigenetic factors that leads to protein mishandling, mitochondrial dysfunction, oxidative stress, excitotoxicity, immune dysfunction, and chronic neuroinflammation [114]. The initial focus on the immune aspect of tic disorders pertained to a subset of tic disorders that start abruptly following a streptococcal infection, which was named “pediatric autoimmune neuropsychiatric disorders associated with group A β-hemolytic streptococcus (PANDAS)” [115]. In the following years, several lines of evidence on immune correlates of tics and TD were explored [5]. First of all, it became clear that the maternal and family history of autoimmune/inflammatory conditions is more common in individuals with tic disorder and TD compared to neurotypical subjects [116]. Among the population in the Swedish National Patient Register, first-degree family members (parents and siblings) of individuals with TD had a higher prevalence of several autoimmune disorders (celiac, thyroiditis, type 1 diabetes mellitus, and psoriasis) [117]. This evidence suggests that an immunogenetic component predisposing to immune dysregulation may be involved in the pathogenesis of the disorder. Further research points to a single-nucleotide polymorphism (SNP) of the TNF gene (-308 A/G) coding for the proinflammatory cytokine tumor necrosis factor (TNF-a) which may be associated with TD [117]. TNF-α plays a crucial role in many inflammatory diseases (asthma, atopic dermatitis, psoriasis, many forms of arthritis, and other autoimmune/inflammatory conditions). GWASs demonstrated positive genetic correlations between allergic and immune/inflammatory-related disorders and many psychiatric disorders, including TD [118]. An association between TD and a lymphocytic gene set driven by variants in the FLT3 gene raises the possibility of neuroinflammation in TS [119]. Many studies have evaluated cytokines in TD subjects, yielding both overlapping and diverging results. This is expected, as the condition fluctuates, and thus, depending on the study design and subgroup studied (see Introduction), the immune profiles vary [120,121,122,123,124,125]. Most of the variability likely reflects the phase of the disease trajectory. Future studies of the immune component of tic disorders/Tourette’s syndrome will need to focus on aligning the timing of sample collection (new onset, relapse, ramping-up phase, peak symptoms, recovering phase, and back to baseline phase). This is likely more feasible in the research subgroup of patients who have post-group A streptococcal escalation of tics followed by resolution.

Another research line has focused on susceptibility to infections (i.e., immunodeficiency) that lead to “frustrated inflammation”. For example, some subjects with TD have lower expression of the toll-like receptor 4 (TLR4) after stimulation with lipopolysaccharide (mimicking a bacterial infection), and higher levels of soluble Cluster of Differentiation (CD) 14, compared to healthy subjects [126]. This pattern may account for the impaired activation of the innate immune response to bacterial components in TD.

On the autoimmune front, a lower number of peripheral regulatory T cells (Tregs) were found in TD subjects compared to controls, which is thought to lead to overactivation of the immune response and predispose individuals to autoimmunity [127]. Although measuring autoantibodies and understanding their impact is always a challenge in human disease, a subgroup of patients with both movement and psychiatric disorders (basal ganglia autoimmunity) suggest that antibodies against cell surface D2 dopamine receptors (D2R) [128] may be involved. Even more interesting is the demonstration that the presence of serum anti-D2R antibody (as determined by a cell-based assay and blinded raters) was significantly associated with tic exacerbation compared to baseline [129].

Immunoglobulin (Ig) subclass deficiency may also play a role in some individuals with TD. Pilot data (from patients with TD and controls) indicate low levels of IgG3, which may be relevant since IgG3 is the most effective IgG in activating complements, thereby playing a critical role in the removal of intracellular pathogens [130]. It is well known that impairment in clearing infections leads to systemic inflammation and autoimmunity, and as mentioned above, population studies link systemic inflammation/autoimmunity to tic disorders [116].

Despite partially inconsistent results regarding cytokines, emerging data support a role for the immune system in the pathogenesis and/or exacerbation of TD and tic disorders. The varying results in cytokine studies may be due to the lack of alignment with regard to disease phase and other study design differences. Consistent with the favorable outcome of the disorder in most patients, a hypothetical immune–inflammatory pathway contribution to pathophysiology may be attenuated in adulthood (due to reduced infection burden in adulthood compared to childhood) and, consequently, inflammatory biomarkers may also be reduced or different.

### 3.5. Intellectual Disability

Intellectual disability (ID) is a neurodevelopmental disorder that begins in childhood and is characterized by intellectual difficulties as well as difficulties in conceptual, social, and practical areas of living, with a prevalence of 2.5% among the general population [1]. Etiology is multifactorial and ranges from genetic to environmental factors. Among these factors, maternal infections during pregnancy are a well-established cause of increased risk of ID in children [131,132]. Beyond the rare occasion of direct injury due to neurotropic infections, the host immune responses to infection and subsequent inflammation are likely a more common mechanism [133].

Over the years, researchers have examined potential immune–inflammatory correlates in individuals with ID [134,135]. For example, studies of participants with 22q11.2 deletion syndrome (22q11.2DS) (a neurogenetic disorder whose phenotype includes both immunodeficiency and ID/psychotic disorder) found higher levels of inflammatory markers (C-reactive protein, IL-6, IL-10, TNFα) compared with healthy individuals [135]. Interestingly, the 22q11.2DS participants with psychotic features had higher levels of IL-6 (*p* < 0.001) and the IL-6/IL-10 ratio (an indicator of a proinflammatory state) compared to the non-psychotic 22q11.2DS individuals; furthermore, IL-6 levels and the IL-6/IL-10 ratio correlated with the severity of cognitive deficits. In summary, individuals with the same genetic condition showed a correlation between severe cognitive and psychotic symptoms and proinflammatory status.

In parallel, environmental factors such as sevoflurane (one of the most commonly used inhaled anesthetics in pediatrics) have been studied because of the potential link to neurotoxicity-induced cognitive impairment [136]. In mouse models, it has been demonstrated that sevoflurane acts through neuroinflammatory pathways. In particular, the expression of IL-17A seems to be upregulated in the hippocampus of sevoflurane-exposed neonatal mice [137], and neuron-localized IL-17A may represent a factor in neuroinflammation and atypical neurodevelopment [138]. Thus, together with the genetic component, an inappropriate inflammatory response to environmental factors (from infectious to toxic exposure) likely plays a significant role in determining and regulating the expressivity of NDDs, including ID. As for the other neurodevelopmental disorders, an abnormal immune response during critical windows of development, along with the resulting abnormal production of neuroinflammatory mediators, can impact brain function and structure.

### 3.6. Schizophrenia Spectrum (SS) and Other Psychotic Disorders (PDs)

The term “autoimmune psychosis” was recently coined to identify new-onset psychosis for which an underlying autoimmune condition is recognized as the driver of the symptoms [139,140,141]. Three subtypes have been hypothesized for this drug-resistant psychosis: (a) psychosis associated with autoantibodies targeting well-characterized synaptic and neuronal cell surface proteins; (b) psychosis associated with classical systemic inflammatory and autoimmune disorders; (c) seronegative but probable autoimmune psychosis (SPAP) [140].

Beyond this rarely diagnosed autoimmune disease, the conditions which are currently labeled as “idiopathic” SS/PD have emerging data supporting the role of neuroinflammation, as indicated by increased levels of inflammatory markers in both serum and CSF [142,143,144]. Data from a large sample of SS/PD patients referred to a tertiary care hospital showed that 45% of patients had at least one CSF abnormality (leukocytosis, elevated protein, elevated albumin quotient, or intrathecal antibody production) and/or an autoantibody [144]. Considering magnetic resonance imaging (MRI) and electroencephalogram (EEG), 75.6% of psychotic patients showed abnormal biomarkers linked to brain inflammation. Different studies have highlighted the persistent elevation of inflammatory markers (TNF-α, IL-1, IL-6, IL-8, IFN-γ, TGF-β) in the sera of schizophrenic subjects [145,146]. Interestingly, IL-1, IL-6, and TGF-β were increased during the first psychotic episode but normalized after antipsychotic treatment, suggesting that they may act as state markers. On the contrary, TNF-α, IL-12, IFN-γ, and soluble IL-2 receptor (sIL-2R) seem to be trait markers, as they remained elevated despite treatment [147]. Elevated levels of both pro- and anti-inflammatory cytokines—TNF-α, IL-1, IL-6, IL-8, IL-1β, and TGF-β—have also been reported in the cerebrospinal fluid of patients [145,148]. Additionally, researchers have found that high levels of inflammatory biomarkers in youth increase the future risk of psychotic disorders [149,150]. Lastly, in the first psychotic episode, the inflammatory biomarkers and the severity of negative symptoms appeared positively related [151].

The increased permeability of the blood–brain barrier during infection and other inflammatory conditions is likely a factor allowing for the penetration of immune cells and inflammatory mediators into the brain and leading to “low-grade neuroinflammation” in a subset of individuals who have been diagnosed with acute psychosis or schizophrenia spectrum disorders [152,153]. Persistent low-grade inflammation may contribute to psychiatric symptoms, behavioral symptoms, and cognitive decline (possibly related to brain injury). This hypothesis is supported by postmortem brain studies of schizophrenia, which indicate elevated inflammatory markers and microglia activation in conjunction with increased expression of proinflammatory genes on both transcript and protein levels [154,155].

In 2020, an international consensus on the diagnostic and therapeutic approach to “autoimmune psychosis” was published [152]. The awareness of the existence of “autoimmune psychosis” has increased in parallel with the identification of autoimmune encephalitis, with prevalent psychotic symptoms sustained by antibodies against synaptic and neuronal cell membrane proteins, such as Anti-N-Methyl-D-Aspartate Receptor (NMDAR) receptor [156,157]. Additionally, several autoantibodies targeting other neuroglial antigens have been identified, such as autoantibodies against AMPAR (α-amino-3-hydroxy-5-methyl-4-isoxazolepropionic acid receptor), CASPR2 (contact-associated protein 2), MOG (myelin-oligodendrocyte glycoprotein), and GABAA/BR (gamma-aminobutyric acid) [29]. Youth meeting criteria for AE [25,27,158] may have a broad spectrum of psychiatric symptoms, including irritability, aggression, inappropriate behaviors, temper tantrums, mood alterations, psychotic symptoms (hallucinations, confabulation, and thought disorganization), memory loss, cognitive impairment, and sleep dysregulation. In youth with NMDAR encephalitis (the most common AE syndrome), 90% develop at least three of the following symptoms within one month of onset: psychiatric symptoms (mentioned above), memory loss, seizures, dyskinesias, a change in the level of consciousness, or automatic dysfunction [158]. Delusion and psychotic symptoms seem to be associated with higher odds for sleep disturbance, oro-facial dyskinesia, and abnormal findings on MRI. In contrast, a waxing and waning presentation of symptoms appears to be associated with abnormal EEG [159].

The term “autoimmune psychosis” has often been considered to refer to atypical, mild, or attenuated forms of classically defined AE [25,158], and it may also pertain to those cases where the patient has clear markers/manifestations of a systemic autoimmune condition in conjunction with atypical psychosis but does not meet criteria for AE. This is especially compelling when both a systemic autoimmune process and psychosis emerge together. Nevertheless, most academic institutions consider AE and autoimmune psychosis to be distinct [152] (with regard to clinical biomarkers and clinical course) but potentially on the same spectrum. Seronegative AE refers to the subgroup of patients who meet AE criteria but do not have an identified autoantibody [25,158], which accounts for nearly half of pediatric AE cases. Immunomodulation is the cornerstone to treating AE and is currently being explored in cases of suspected autoimmune psychosis which do not meet criteria for AE [140,160], inferring the diagnosis according to the “ex juvantibus” criteria. Remarkably, in seronegative autoimmune psychosis, neuropsychiatric features with psychotic symptoms appear dominant [140,161]. Finally, epidemiological studies are consistent in demonstrating a considerably higher lifetime prevalence of autoimmune diseases in people with psychosis compared to the general population [162]. Data from a Danish population-based study showed an increased risk of subsequent diagnosis of autoimmune disease in patients with schizophrenia, possibly reflecting neuropsychiatric manifestations of smoldering autoimmunity and/or shared immunogenetic risk factors [163]. In parallel, infections have been associated with the development of schizophrenia [164,165] regardless of the nature of the pathogen. Infections are considered a potential trigger for autoimmune diseases. Interestingly, when autoimmune diseases and severe infections co-occur, the risk of schizophrenia significantly increases [166]. Patients with autoimmune psychosis appear to be poor responders to antipsychotic agents [9], while they often respond to anti-inflammatory treatment [167]. Published data support the hypothesis that the immune system and its related inflammatory response are intensely involved in the pathogenesis of a subset of patients with SS/PD, which are now categorized as having “autoimmune psychosis” or AE.

### 3.7. Obsessive–Compulsive Disorder

OCD is a severe and common mental illness with a lifetime prevalence of 1–3% [168] and driven by an imbalance of cortico-striato-thalamocortical circuits and alterations in serotonergic, glutamatergic, and dopaminergic neurotransmission, modulated by genetic and epigenetic factors [169]. Recently, the immunoinflammatory etiopathogenetic hypothesis has been considered due to the higher occurrence of autoimmune and infection-related conditions compared to the general population, as well as evidence of neural antibodies, microglial dysregulation, inflammatory markers, and persistent low-grade inflammation in a significant subset of patients [170,171]. In a subgroup of OCD patients (pre-pubertal, abrupt onset, relapsing remitting), autoimmunity may be driven by shared surface epitopes between streptococcal proteins and neurons [172]. This assumption led to the formulation of the concept of Pediatric Autoimmune Neuropsychiatric Disorder Associated with Streptococcal infection (PANDAS), a reactive inflammatory syndrome with OCD symptoms that abruptly start, remit, and relapse (in vulnerable individuals) in the setting of Group A-beta-hemolytic Streptococcus (GABHS) infection [115]. Extensive population-based cohort studies confirmed an association between GABHS infection and a risk of developing OCD [173]. Furthermore, IgG antibodies from children with PANDAS bind to cholinergic interneurons (CINs) in the striatum of mouse brain slices [174], a critical cellular target whose ablation is associated with OCD symptoms and tic-like stereotypies [175].

The MIA model has also been hypothesized for OCD but not validated. In a large Swedish cohort, prenatal maternal (but not paternal) infection and early childhood infection increased the risk of OCD and TD, but this was not significant after controlling for genetic vulnerabilities (using sibling data) [176]. OCD symptoms are also very common in autoimmune encephalitis, both in adults [139] and in children [177]. Furthermore, OCD can accompany well-established CNS autoimmune disorders, such as multiple sclerosis [178] or systemic autoimmune diseases, such as systemic lupus erythematosus, dermatomyositis, and Sjögren’s syndrome [179,180,181]. Hence, some authors have proposed to define the concept of “autoimmune OCD” subtype [182], in the context of the so-called “secondary OCD”, corresponding to the DSM-5 diagnosis of” Obsessive-Compulsive and Related Disorder Due to Another Medical Condition” [1].

Inflammatory markers and immunogenetic predisposition were evaluated in assumed “primary forms of OCD”. For example, a meta-analysis demonstrated that serum and CSF anti-basal ganglia antibody (ABGA) levels were fivefold increased in individuals with primary OCD compared with controls, and comparable to TD, ADHD, and PANS patients [183]. A meta-analysis of plasma serum levels of proinflammatory cytokines reported a significant reduction in IL-1β levels in individuals with OCD compared to controls, a decrease in IL-6 levels in studies involving children, and elevated TNF-α levels in studies including subjects with comorbid depression [184].

Considering links between infections, autoimmunity, and psychiatric disorders, it is not surprising that the human leukocyte antigen (HLA) system is implicated in all psychiatric disorders, including OCD [185]. An Exon-focused genome-wide association study in OCD patients showed that two distinct HLA regions represented shared polygenic risk factors with schizophrenia [186]. A robust link has been found between early-onset OCD and HLA-class II, particularly with the allele HLA-DRB1-04, involved in autoimmune type-1 diabetes [187,188].

Finally, immunogenetic determinants are being explored. A meta-analysis on the TNF-α-238G/A gene polymorphism demonstrated its association with a decreased risk of OCD susceptibility [189]. Additional studies suggest that polymorphisms in the progranulin gene (PGRN), a key regulator of brain inflammation, are associated with an increased risk of developing OCD and interact with childhood trauma [190].

Overall, the current literature seems to support a connection between immune-related conditions and OCD (through immunogenetic predisposition combined with environmental factors), with a subgroup of patients likely having an inflammatory driver of OCD.

### 3.8. Pediatric Acute-Onset Neuropsychiatric Syndrome

PANS is a recently described syndrome characterized by the acute onset of obsessive–compulsive symptoms and/or a severe food intake restriction, associated with at least two other acute-onset severe symptoms (cognitive, behavioral, or affective symptoms such as irritability, anxiety, emotional lability, behavioral regression, urinary frequency and/or enuresis, sensory or motor alterations, and sleep disturbances). In addition to urinary changes, patients may experience other autonomic symptoms, such as mydriasis and postural orthostatic tachycardia [191,192,193,194,195,196,197]. A significant percentage of PANS patients also experience psychotic symptoms, such as auditory and/or visual hallucinations, thought disorganization, and delusions, and tend to have a regressive course or more severe long-term impairment [198,199,200]. Current evidence suggests that PANS may represent a post-infectious systemic inflammatory condition that involves complement activation [201] and neural autoimmunity [202]. It is associated with the development of arthritis and other systemic autoimmune conditions [203]. The condition may also involve proinflammatory monocytes [204] and activation of the hypothalamic–pituitary–adrenal (HPA) axis during stress, which further exacerbates neuroinflammation [205]. Although PANS is thought to be a post-infectious condition, the PANS clinical diagnosis is agnostic to the preceding infection, as causality cannot be proven at the individual level, and many patients present after the window of opportunity to diagnose GABHS infections. While the PANDAS diagnosis is helpful and critical for research (since it requires documentation of GABHS with two deteriorations), the PANS diagnostic criteria were intended for clinical care and overlapped with the PANDAS criteria. While other infections may precede the PANS condition, the link between OCD and other infections has not been demonstrated on an epidemiological level. However, convincing cases of PANS following SARS-CoV-2 have been published [206,207,208], which may also include reactivation of herpes viruses, further contributing to neuroinflammation [209].

Evidence for basal ganglia involvement in this inflammatory condition includes four imaging studies [210,211,212,213], four sleep studies indicating movements/loss of atonia during REM sleep [194,196,197,214], neurological soft signs pertaining to the basal ganglia [215], and autoantibodies that target cells in the basal ganglia [174,202,216,217]. Consistent with preclinical studies suggesting a crucial role of T-helper-17/interleukin-17 (IL-17) inflammatory-mediated response in the pathogenesis of PANDAS/PANS [33,35], a recent study on children with PANDAS/PANS found increased serum and CSF levels of IL-17 compared to healthy children, with a trend of increasing levels in post-pubertal children and higher concentrations in the CSF than in serum [218]. Beyond IL-17, higher levels of various cytokines and inflammatory molecules have been found in PANDAS/PANS, with both overlapping and diverging results (which is expected given that the phase of illness impacts findings and that study design differences will yield different findings, as described previously) [219]. IL-1-β and TNF-α were elevated in a cohort of children with PANS, both in the remitting and chronic course groups [220].

Observational and case–control studies have found several general inflammatory biomarkers in PANDAS/PANS, including complement activation (high C4a and low C4), leukopenia, increased C-reactive protein, Neuron-specific Enolase (NSE), and serum amyloid A (SAA) [50,205,220,221,222]. As expected, low-level immune activation indicators were found, such as positive antinuclear antibodies, elevated thyroid antibodies, antineuronal antibodies (e.g., dopamine receptors D1 and D2, lyso-ganglioside-GM1, CaMKII-activity, and β-tubulin antibodies), and positive celiac tests [50,193,203,205,220,222,223,224]. Similar to other NDDs, a family history of autoimmune/inflammatory disorders is very common in PANS [50,222,225,226,227], such as multiple sclerosis, Systemic Lupus Erythematosus (SLE), Crohn’s disease, rheumatoid arthritis, Hashimoto’s Thyroiditis, rheumatic heart diseases, and Sydenham chorea. Autoimmune conditions seem to be common (20%) in mothers of children with PANS [198], highlighting the importance of a genetic propensity for immune dysregulation. Additionally, patients with PANS have a high rate of developing juvenile arthritis (typically enthesitis-related arthritis but not rheumatoid or systemic-onset arthritis) and other autoimmune diseases [203]. A whole-exome (WES) and whole-genome sequencing (WGS) study [228] identified several genetic factors underlying some cases of PANS. Ultra-rare variants in 11 genes (PPM1D, SGCE, PLCG2, NLRC4, CACNA1B, SHANK3, CHK2, GRIN2A, RAG1, GABRG2, and SYNGAP1) were found, suggesting that some cases may have genetic variants that contribute to the modulation of peripheral immunity and microglia, as well as BBB integrity and the enteric nervous system. Interestingly, most of these genes regulate both the immune response and synaptogenesis and are involved in the etiology of NDDs. No specific studies are available on MIA in PANS [229,230]. However, the WES and WGS study cited above [228] detailed that the genes found in the patients with PANS had expression in the choroid plexus and brain vasculature, which can be linked to MIA. Reliably, transcriptome analysis suggests that activation of innate immune pathways may be the link between maternal inflammatory state and the future development of tics and OCD in offspring [19]. Similar to NDDs, the liability for developing PANS could begin during fetal life in genetically predisposed individuals.

## 4. Discussion

**Figure 2:**
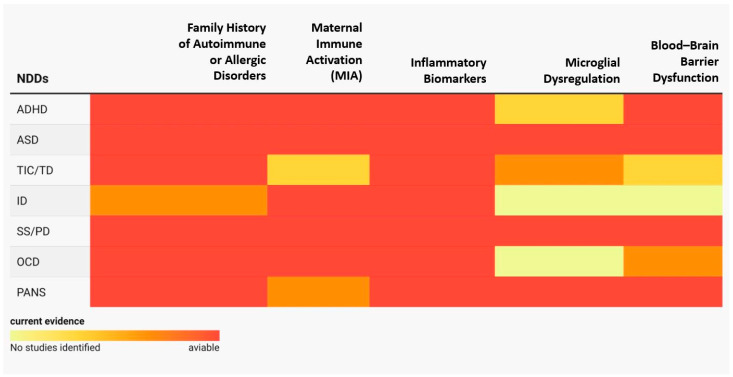
A heat map summarizing the strength of immuno-inflammatory evidence across major neurodevelopmental disorders (NDDs) and Pediatric Acute-Onset Neuropsychiatric Syndrome (PANS). Evidence was extracted from Table 1 and graded on a 0–3 scale: 0 = no studies identified; 1 = limited or indirect evidence; 2 = moderate/conflicting evidence; 3 = strong or consistent evidence.

This comprehensive literature review aims to look at neurodevelopmental disorders (NDDs) and PANS from an immunological point of view and provide an integrated perspective. Table 1 summarizes the main evidence of the shared immune–inflammatory correlates between the NDDs, TIC/TD, ID, OCD, SS/PD, and PANS. The current evidence supports a common framework and likely shared pathogenic mechanisms among these NDDs and PANS. This review offers a synthesis and a comprehensive interpretation of immunological data, immunogenetic data, and family correlates, providing substantial evidence supporting the role of the immune–inflammatory responses in brain development and function (Figure 2).

Brain transcriptome studies and epigenetic analyses of individuals with NDDs have demonstrated dysregulated immune pathways [102]. Most of the genes regulating the immune response are also involved in synaptogenesis, suggesting shared factors between the CNS and the immune system. The co-occurrence and familial clustering of neurodevelopmental disorders and immune disorders indicate that the genetic substrate is shared mainly between the NDDs and autoimmune diseases [231], as described both for NDDs and PANS. Moreover, many nationwide studies on the risks of multiple mental disorders among the offspring of parents with autoimmune disorders demonstrated strong correlations. For instance, an increased risk of ASD, ADHD, bipolar disorder, and major depressive disorder has been associated with rheumatoid arthritis (RA), with higher risks in children of mothers with RA rather than of fathers with RA [232], supporting a role for maternal immune activation in neural development. Recently, an umbrella review [233], encompassing around 12.5 million participants, confirmed the co-occurrence of systemic diseases and ADHD, one of the main NDDs. It measured the strength of associations between ADHD–asthma and ADHD–obesity and depression–asthma, as well as between dermatitis and ASD/ADHD, asthma and anxiety/ASD/depression/bipolar disorder, obesity and ADHD/ASD/depression, revealing a strong association between a broad range of mental disorders in youth and suggesting that these conditions are linked to an activated immune system.

A key research field is focused on the prenatal deviation in neurodevelopmental processes due to the interaction between immune factors and the CNS. Glial cells are altered during neuroinflammatory processes and may influence brain development and function. Microglia have been suggested to be the “crucial architects of the developing brain” [37] for their role in regulating a plethora of neurodevelopmental processes [44,234]. Furthermore, the influence of and interaction between epigenetic and neuroinflammatory factors in the regulation of microglia activity is now recognized to be a major etiopathogenetic mechanism of neurodevelopmental disorders. Animal models of MIA have revealed long-term alterations in the brain structure and immune system of offspring [23] including persistent proinflammatory microglial activity and a continuous influence on the formation and maintenance of neuronal networks. Overall, proinflammatory maternal activation represents one of the primary mechanisms through which the immune system guides neurodevelopment, with an overwhelming immune reaction leading to altered CNS development and potential impacts on long-term functioning [10,89]. However, systemic inflammation (e.g., infectious or autoimmune diseases) later in life also contributes to microglial shifts from their typical protective anti-inflammatory function/profile to a proinflammatory profile [46]. Therefore, microglia may also play a key role in synaptic pruning during adolescence and in maintaining the homeostasis of synaptic circuits in the CNS throughout the whole life span. Through receptors for purines (such as adenosine triphosphate) and common neurotransmitters (such as glutamate), microglia mediate local neuronal activity and regulate the activation rate of neurons through the release of extracellular vesicles or signaling molecules, such as tumor necrosis factor (TNF). In this way, microglia induce synaptic plasticity and sustain functions such as motor learning and memory [36,235].

**Figure 3:**
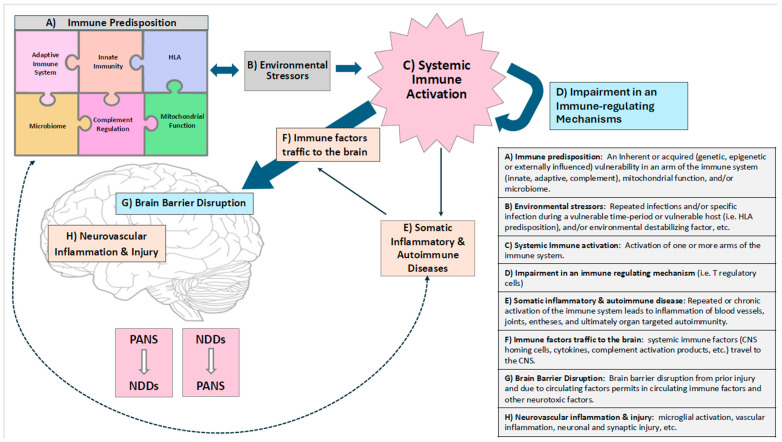
PANS: a syndromic constellation overlapping with other NDDs. Legend: NDDs: Neurodevelopmental Disorders; PANS: Pediatric Acute-Onset Neuropsychiatric Syndrome.

Environmental factors may have a proinflammatory role in CNS maturation by way of epigenetic modifications [38]. For example, prenatal alcohol exposure seems to alter the immune system through epigenetic changes that lead to permanent modification of the microglial activation state, resulting in a long-lasting imbalance between the immune system and CNS [236]. In this complex interplay between genetic and epigenetic forces that influence the immune system and CNS, neuroinflammation represents a point of intersection (Figure 3). As this review underlines, this is true for all neurodevelopmental disorders. Therefore, neuroinflammation becomes a possible common denominator of the whole spectrum of NDDs and PANS, since PANS shares with NDDs both the pathogenetic determinants and the clinical phenomenology [237].

The PANS syndromic constellation includes symptoms that largely overlap with those of all other NDDs. Furthermore, PANS has been described in both neurotypical children and subjects with neurodevelopmental disorders [39]. Pre-existing neurodevelopmental disorders or, at least, some degree of underlying neurodevelopmental abnormality has been described in 22–71% of PANS cohorts [192,222,225]. This leads us to postulate a possible relationship between PANS and NDDs for which the causality and directionality link should be established. Does a pre-existing NDD create vulnerability to a PANS-like inflammatory episode? Can a PANS-like process in utero or early childhood manifest as a classic NDD phenotype? The conceptual construct of MIA may offer an explanatory model. In an MIA model, individuals who have had a “first hit” during intrauterine life will be more vulnerable to “second hits” (e.g., infections, stress, drug abuse) met at successive ages, with a resulting heightened risk of developing psychiatric syndromes [23]. This model appears highly consistent with the waxing and waning symptom patterns of many NDDs (e.g., autism, tic disorder). In individuals with a further genetic propensity to experience significant inflammation after Group A Streptococcus or other infections, it may be expected that this subgroup of patients with NDDs will meet the criteria for PANDAS/PANS. Very often, these “flares” will occur with an increase in pre-existing symptoms (e.g., hyperactivity, impulsivity, repetitive behaviors, etc.). Additionally, symptoms belonging to the typical PANS syndromic constellation (obsessions, food restriction, irritability, anxiety, sleep disturbances, autonomic symptoms) also overlap with the pre-existing symptoms. These “flares” may be transient or may lead to a permanent reduction in functional level. They may also follow an infectious disease or an environmental stressor, as it happens in children with a new onset of PANS not preceded by pre-existing NDDs. Consistent with this hypothesis, outcome studies on children with PANS, although still very limited, suggest that sustained recoveries are rare, and both persistent symptoms and frequent relapses are common [39,238]. In a two-to-five-year follow-up study on the Karolinska PANS cohort, out of 32 patients with no pre-existing NDDs, 13 (38%) received a neuropsychiatric diagnosis. Nine (26%) were diagnosed with ADHD, three (9%) with ASD, and one (3%) with intellectual disability, despite a significant reduction in clinician-rated PANS symptom severity and an improvement in the general function.

The scientific community is attempting to establish the existence of PANS as a distinct nosological entity [191,222] through the application of statistical approaches [50]. However, the symptoms appear poorly specific (except for the criteria specifying “abrupt” onset) and largely overlap with neurodevelopmental disorders, OCD, and psychotic disorders. The growing recognition of the significant phenotypic overlap between NDDs has led to a shift away from a categorical approach towards a more comprehensive perspective, incorporating the concepts of “spectrum” [239] and “dimensions” [240]. The Research Domain Criteria (RDoC) project by the National Institute of Mental Health drove clinical research toward this direction, describing dysfunctional behavioral phenotypes at many levels of information, including genomics, circuits, and physiology [241]. The PANS diagnostic construct includes “dimensions” largely shared with NDDs in the affective domain (e.g., irritability, emotional lability, separation anxiety) as well as in the behavioral, cognitive, and motor domains (e.g., hyperactivity, attention deterioration, repetitive thoughts/actions, vocal and motor tics, sensory disorders, sleep alterations). At the same time, at a neurophysiological level, areas and circuits of disturbed neural activity, often within the basal ganglia, cerebellum, and prefrontal cortex, have been described for PANS as well as for NDDs [210,242]. Finally, at a genomic level, a group of altered genes regulating both the immune system and synaptogenesis (PPM1D, SGCE, PLCG2, NLRC4, CACNA1B, SHANK3, CHK2, GRIN2A, RAG1, GABRG2, SYNGAP1) seems to play a multifactorial and probabilistic role in PANS along with NDDs [228].

Accordingly, PANS could represent a transnosographic syndromic constellation that precedes and/or overlaps neurodevelopmental disorders (Figure 3). Most NDDs have a fluctuating course with flare-ups and recoveries. For all these reasons, PANS could be considered a neurodevelopmental construct that explains some of the mechanisms that support neurodevelopmental disorders in the context of the etiological and phenotypic complexity that characterizes NDDs and psychiatric disorders [39]. Transcending a rigid categorical approach allows us to conceptualize PANS either as a transnosographic condition common to all NDDs or as a potential precursor, depending on the timing of onset (Figure 3). In any case, PANS is linked to inflammation-based pathogenic factors largely shared with NDDs. Therefore, PANS may illustrate how psychiatric symptoms can arise from a complex interaction of diverse biological mechanisms and clinical manifestations.

In summary, human and animal models of autoimmune psychiatric diseases support a multiple-hit hypothesis in which psychiatric symptom onset coincides with an infection and/or environmental stressor that breaks the immune self-tolerance [243]. Then, neuroinflammation is produced according to the genetic liability to inflammation/autoimmunity in a subset of individuals. Finally, a systemic inflammatory response, likely involving both innate and adaptive immune mechanisms, drives the entry of CNS-homing cells, cytokines, and autoantibodies into the CNS, activating the inflammasome/microglial cells, as well as other immune mechanisms [33,35]. This pathway might not be specific for AE and PANS and might represent the common denominator of multiple disorders. It might interfere in a very early stage of neurodevelopment and lead to syndromic entities that we define as neurodevelopmental disorders.

### Forthcoming Issues

The strong genetic correlations between NDDs, PANS, and immune disorders and the identification of shared biological mechanisms and conditions of susceptibility suggest that we must act at multiple levels. Primarily, we must develop preventive interventions aimed at reducing the exposure to risk factors starting from pregnancy. Secondarily, we must act through specific treatments in the later stages of life. The effectiveness of preventive strategies at a population level aiming to optimize maternal health during pregnancy and early infant health should be seen as a priority, especially in the first 1000 days of life [244]. Thus, preventive interventions and monitoring activities should be organized for pregnant women, especially if they have immune–inflammatory disorders or a high familial risk of having offspring with NDDs. Studies on neuroinflammation have also investigated the role of the gut–brain axis involving a microbial–immune–neuronal relationship in NDDs [245]. However, a paucity of studies has been conducted on microbiota composition in children with PANS. A larger investigation of potential mechanisms involving the gut microbiota and its effects on immune modulation, metabolic regulation, and neurotransmitter modulation is needed.

Since metabolic processes play a critical role in immune regulation, metabolomics could represent an available method to evaluate immune response and inflammatory status. A few studies provide a systematic analysis of metabolites in PANS, showing a peculiar plasma metabolic profile [246]. Several molecules, such as 2-hydroxybutyrate, glycine, glutamine, histidine, tryptophan, phenylalanine, and tyrosine, had significant differences compared with healthy controls, accounting for alterations in specific patterns of neurotransmission, neuroinflammation, and oxidative stress. A larger number of studies are available in ASD describing alterations in amino acid and energetic pathways [247]. A unique study compared the metabolomic pathways of children with ASD and PANS [248], showing a decrease in glycine and asparagine concentrations as a shared biomarker of ASD and PANS, potentially leading to detrimental effects on inflammatory homeostasis and suggesting an “inflammatory signature” for both groups.

Inflammatory processes could account for the inadequate response to anti-psychotic treatments in a subset of patients with psychosis. The same issue could be true for the early stages of OCD, ADHD, ASD, SS/PD, and PANS.

Thus, it is mandatory to find new diagnostic approaches aimed at verifying if an immune–inflammatory etiopathogenesis underpins a set of neuropsychiatric symptoms. There is still a paucity of randomized controlled trials (RCTs) and a lack of evidence of the valuable effects of immune-based interventions. Starting from the evidence discussed in this review, finding a treatment addressing the numerous factors and effects linked to a brain inflammatory state could represent a challenge. It should be modulated on the state of the disease (acute, chronic, fluctuating), on the specific immune profile (overactive, immune deficiency, dysregulated), on potential circulating biomarkers (e.g., antibodies, cytokines, chemokines, microRNAs, neuropeptides), on possible brain alterations (e.g., damaged BBB, activated microglia, dysregulated neurotransmission), or likely on all of these factors at the same time. Further observational studies, together with the results from preclinical research, will inform and optimize RCT trial design aimed at establishing the most appropriate treatment strategies. RCTs should be organized to clarify whether conventional therapies might be augmented with new treatment strategies aimed at modulating the systemic immune response and/or neuroinflammation in cases where there is reasonable suspicion of an immune–inflammatory etiopathogenesis. Clinical translational studies should be organized to confirm experimental models that explain that chronic neuroinflammation can injure the brain, thus driving maladaptive symptoms and behaviors.

Furthermore, RCTs should be conducted to verify if immunomodulating agents administered in the early stages of the immune–developmental and immune–psychiatric illnesses can avoid “organ failure”, which otherwise inevitably leads to permanent cognitive and functional impairments. To achieve all of these purposes, both a paradigm shift and a cultural change in clinical medicine are necessary. For a plethora of reasons (e.g., late discovery of lymphatic vessel into the brain, idea of brain inaccessibility by the BBB, Cartesian dualistic view of mind and body, etc.), there is a cultural delay in accepting that the brain is an organ like all others that is susceptible to injury by the immune–inflammatory cascade. Analogous to the management of inflammatory diseases affecting organs such as the kidneys, heart, and liver, it appears important to consider comparable approaches and strategies for neuroinflammatory conditions involving the brain.

## 5. Limitations

This scoping review has several limitations inherent to the methodological framework of scoping studies. It was not designed to provide a quantitative synthesis of the evidence. Consequently, the findings should be interpreted as a broad mapping of the existing literature rather than a basis for definitive conclusions on effectiveness or causality.

A further limitation lies in the use of only two electronic databases (PubMed and Web of Science). Although these platforms offer extensive and multidisciplinary coverage, relying exclusively on them may have led to the omission of relevant studies indexed in other databases. To address this limitation, we have also implemented a handmade search and broadened the search criteria. On the other hand, the wide scope of the initial search strategy might also represent a limitation. While this approach was selected to maximize sensitivity and reduce the risk of missing relevant research, it resulted in a very large number of records, potentially introducing noise and decreasing the specificity and accuracy of the review conclusions. To minimize secondary bias, rigorous screening procedures were employed to enhance the relevance and reliability of the final selection. Lastly, this review included only articles published in English, Italian, or Spanish, or those for which a full translation was available. This language restriction may have introduced selection bias and resulted in the exclusion of potentially relevant studies published in languages other than English.

Finally, from a conceptual point of view, this review presents a unique explanatory model, neglecting to take into consideration alternative interpretations. For example, could these shared immune findings simply be non-specific epiphenomena of chronic CNS dysregulation, rather than a core pathogenic driver? What is the role of molecular and cellular events related to neurotoxicity and oxidative stress in the potential development of negative outcome pathways? Does neuroinflammation represent a key causal event or only a final common pathway of several pathogenic agents? Answering these questions should be a priority for the scientific community due to the chief implications on therapeutic choices.

## 6. Future Directions

Several gaps in the current literature have emerged. These include inconsistent use of diagnostic criteria, limited longitudinal data, underrepresentation of pediatric ID and OCD in immune–psychiatric research, and a lack of controlled trials assessing immunomodulatory treatments. Moreover, few studies adopted a transdiagnostic framework to compare immune profiles across diagnostic categories. To fill this gap, several promising directions for future research can be pointed out from the examined data. First, longitudinal studies are needed to clarify the temporal relationship between the onset of PANS and the development or exacerbation of NDDs. These studies could help disentangle causality and identify critical periods for intervention. Second, research should further explore the several neuroimmune mechanisms that may link PANS and NDDs, particularly through the study of inflammatory markers, autoimmunity, and blood–brain barrier dysfunction.

Furthermore, controlled clinical trials assessing the efficacy and safety of immunomodulatory treatments in pediatric populations with overlapping PANS and NDD features are warranted. To enhance the design of future randomized controlled trials, patient stratification based on clinical phenotype (e.g., severity, age of onset, presence of comorbid NDDs) and immunological profile may be critical. Moreover, the selection of standardized and multidimensional outcome measures—encompassing both core neuropsychiatric symptoms and biomarkers of neuroinflammation—would improve the interpretability and clinical relevance of findings. Incorporating follow-up assessments over time will also help determine the durability of treatment effects.

Finally, future studies would benefit from standardized diagnostic protocols and larger, multicenter samples to improve replicability and comparability of findings. These efforts would contribute to a more integrated understanding of PANS as a potentially transnosographic or precursor condition within the NDD spectrum.

## 7. Conclusions

This review makes a unique contribution to the field by integrating evidence on immune-related mechanisms across traditionally distinct neuropsychiatric disorders within a neurodevelopmental framework. The findings support the relevance of both environmental and inflammatory factors in influencing brain development and, consequently, the developmental trajectory. Among these factors, both the innate and adaptive immune system play a crucial role in genetically susceptible populations. This review improves the awareness of immune/neuroinflammatory correlates supporting NDDs. Furthermore, it suggests adopting a transnosographic approach to neuropsychiatric disorders, including PANS as a syndromic construct that overlaps with NDDs. Further research is needed to identify both preventive strategies for the protection of fetal brain development and postnatal treatments in mitigating the risk of developing NDDs.

## Figures and Tables

**Table 1:** **A summary of immune and inflammatory alterations across neurodevelopmental and neuropsychiatric disorders.** This table synthesizes the available evidence for each condition across six immunological domains: (1) genetic immune-related alterations, (2) familial autoimmune history, (3) maternal immune activation (MIA), (4) inflammatory biomarkers, (5) microglial dysregulation, and (6) blood–brain barrier (BBB) dysfunction. Each cell reflects the strength or type of evidence: **Yes** (current evidence available), **Indirect evidence**, **Limited evidence**, **Evidence not available**, or **No studies identified.** Arrows indicate the direction and intensity of biological level changes reported in the literature: ↑ = increased levels; ↓ = decreased levels.

NDDs	Blood–Brain Barrier Dysfunction	Microglial Dysregulation	Inflammatory Biomarkers	Maternal Immune Activation (MIA)	Family History of Autoimmune or Allergic Disorders	Genetic Immune-Related Alterations
**ADHD**	**Yes**: In SHR models, chronic inflammation and altered autophagy impair BBB integrity, with reduced expression of tight junction proteins such as **occludin** and **ZO-1** [229].	**Limited evidence:** Prenatal immune activation and antibody response against dopaminergic structures suggest early microglial dysfunction, affecting cortical development and synaptic dopamine regulation [66,69].	**Yes**: ↑ **IL-6, TNF-α, and CRP,** and the presence of autoantibodies against basal ganglia and dopamine transporters. The imbalance between pro- and anti-inflammatory cytokines correlates with clinical severity [69,70,71].	**Yes**: In mouse models, MIA reduces prefrontal cortex volume and alters the development of dopaminergic neurons via dysregulation of the Sonic Hedgehog (SHH) pathway [66]. In a Danish mother–child cohort, **↑ maternal hsCRP** levels during pregnancy were associated with an increased risk of ADHD at age 10 [68].	**Yes**: The prevalence of inflammatory and autoimmune diseases such as **asthma, eczema, rheumatoid arthritis, psoriasis, type 1 diabetes**, and **IBD** among relatives—especially mothers—of individuals with ADHD [57,61,125].	**Yes**: Polymorphisms in genes involved in the inflammatory response, such as **TNF-α, IL-1β, and IL-6** [62]. GWASs confirmed the association with genes regulating the inflammatory cascade [63,64].
**ASD**	**Yes**: Defects in amino acid transport across the blood–brain barrier have been linked to impaired neurological development in ASD [230].	**Yes**: Postmortem studies show microglial and astrocyte activation in cortex, white matter, and cerebellum, linked to neuroinflammation and synaptic dysregulation. MIA triggers brain inflammation, oxidative stress, and synaptic ultrastructural changes [101,103,104,107].	**Yes**: **↑** maternal and neonatal levels of **IL-6, IL-4, IL-21, TNF-α, BAFF, CCL2**, and **CXCL8**, associated with altered amygdala connectivity and later cognitive impairments [93,96,97,108].	**Yes**: Infections, **asthma, maternal obesity, thyroid autoimmunity** (e.g., TPO-Ab), and elevated CRP during pregnancy increase the risk of ASD in offspring [68,88,89,90,92,95].	**Yes**: Familial co-occurrence of **allergies, autoimmune diseases, and psoriasis** in parents of individuals with ASD, especially mothers. One study reported a significantly increased risk with combined maternal asthma and obesity [89,90].	**Yes**: ↑ autoantibodies, immunoglobulins, and peripheral cytokines; ↓ regulatory T and B cells. Polymorphisms and abnormalities in genes related to immunity and inflammation, including **TPO-Ab** and immune signaling proteins [90,109,111].
**TIC/TD**	**Evidence not available**: Preliminary evidence suggests that systemic inflammation may impair the BBB, but further studies are needed [5].	**Indirect evidence:** Studies showing reduced regulatory T cells (Tregs) and altered innate immune responses suggest microglial activation as an inflammatory mediator [5,126,127].	**Yes**: ↑ levels of **TNF-α, IL-6, IL-12p40, IL-4,** and **IL-8** correlate with tic severity. ↑ **anti-D2R** autoantibodies during clinical exacerbations [122,123,129].	**Limited evidence**: Prenatal immune activation is hypothesized as a trigger of alterations in the neural circuits underlying tics [19,176].	**Yes**: **↑** prevalence of **rheumatoid arthritis and lupus** in first-degree relatives of individuals with TD [116].	**Yes**: SNP in the **TNF-α gene** (-308 A/G) associated with TD. GWASs show correlations with immune-related variants (FLT3), HLA system, and **IL-1α** [116,117,119].
**ID**	**No studies identified**: No direct data are available on BBB alterations in individuals with ID.	**Evidence not available:** No direct evidence of microglial involvement in individuals with ID.	**Yes**: Individuals with ID (including syndromic forms) show altered cytokine profiles, with ↑ levels of **IL-6, TNF-α, IL-10**, and CRP. Systemic inflammation has been correlated with cognitive severity [134,135].	**Yes**: Maternal infections during pregnancy increase the risk of ID in the child. Beyond direct damage from infectious agents, maternal immune activation plays a key role through the production of proinflammatory cytokines [131,132,133].	**Indirect evidence**: No consistent data available on familial autoimmunity in non-syndromic forms. However, in certain syndromic forms (e.g., Down syndrome, 22q11.2DS), a shared immunogenetic component has been hypothesized [135].	**Indirect evidence**: Evidence from individuals with 22q11.2 deletion syndrome shows that a higher IL-6/IL-10 ratio correlates with cognitive and psychotic symptom severity. While not directly studied in idiopathic schizophrenia, this supports the hypothesis of immune involvement [135].
**SS/PD**	**Yes**: Neurovascular inflammation and increased BBB permeability facilitate the entry of immune mediators into the brain, contributing to chronic neuroinflammation [152,153].	**Yes**: Postmortem evidence of activated microglia and upregulation of proinflammatory genes in individuals with schizophrenia [154,155].	**Yes**: Elevated serum and CSF levels of **IL-6, IL-1β, IL-12, TNF-α, TGF-β,** and **IFN-γ**. Some cytokines (e.g., IL-6, IL-1β) are increased only during acute episodes, while TNF-α, IFN-γ, and sIL-2R remain persistently elevated (state vs. trait markers) [142,147,148].	**Yes**: Maternal infections and severe early-life infections increase the risk of schizophrenia. The risk is further amplified when autoimmune diseases are also present [164,165,166].	**Yes**: The risk of schizophrenia is significantly increased in individuals with a personal or **family history of autoimmune diseases** [162,163].	**Yes**: HLA polymorphisms (e.g., HLA-DQB1) are associated with schizophrenia risk and immune dysregulation. Additional associations have been described between schizophrenia and alleles linked to autoimmune encephalitis (e.g., NMDAR) [156,186].
**OCD**	**Indirect evidence:** Not directly reported. However, in individuals with neuronal antibodies (e.g., PANDAS), altered BBB permeability has been hypothesized to allow for IgG entry into the brain [174].	**No studies identified**: No direct evidence specific to OCD, but microglial involvement is hypothesized in autoimmune-related subtypes such as PANDAS, which share overlapping features.	**Yes**: ABGAs (anti-basal ganglia antibodies) are up to 5 times higher in OCD patients compared to controls. Some studies report **↑ TNF-α and ↓ IL-1β** and **IL-6**, especially in children [183,184].	**Yes**: Prenatal or early childhood infections increase the risk of OCD, but not paternal or sibling infections, suggesting vulnerability linked to early immune exposure and genetic predisposition [176].	**Yes**: Frequently co-occurs in individuals with **lupus, multiple sclerosis, and dermatomyositis**, suggesting a possible shared autoimmune background in predisposed families [179,180,181].	**Yes:** GWASs identified two HLA regions associated with OCD risk, shared with schizophrenia. The HLA-DRB1*04 allele has been linked to early-onset OCD. Alterations in the **PGRN gene (progranulin),** have also been reported in patients with OCD and a history of childhood trauma [186,187,190].
**PANS**	**Yes**: Genetic variants in vascular genes and increased cytokines suggest heightened BBB permeability. The presence of IL-17 in CSF supports the hypothesis of central immune infiltration [35,228].	**Yes**: **Th17/IL-17** response suggests active neuroglial inflammation. Direct microglial involvement has been observed in neuroinflammation and synaptic dysregulation [33,35].	**Yes**: Elevated serum and CSF levels of **IL-1β, IL-6, TNF-α,** and **NSE** have been reported in PANS patients, along with increased **CRP, SAA, and complement activation** [220,221].	**Indirect evidence:** No direct studies exist, but candidate genes for PANS are expressed in the choroid plexus and cerebral vessels, supporting a possible early role of MIA in vulnerability [19,228].	**Yes: ↑** About 70% of individuals with PANS have a family history of autoimmune disorders. Mothers show a **20% prevalence of severe autoimmune diseases,** suggesting genetic predisposition to autoimmunity [50,198,225].	**Yes**: WES/WGS studies identified ultra-rare variants in **11 genes (e.g., PPM1D, SYNGAP1, NLRC4, SHANK3, RAG1)** involved in immune response, synaptogenesis, blood–brain barrier integrity, and the enteric nervous system [228].

Legend: ID: intellectual disability; ASD: autism spectrum disorder; ADHD: attention deficit/hyperactivity disorder; TIC/TD: tic and Tourette’s disorder; OCD: obsessive–compulsive disorder; SS/PD: schizophrenia spectrum and other psychotic disorders; PANS: Pediatric Acute-Onset Neuropsychiatric Syndrome.

## Data Availability

The data presented in this study are available on request from the corresponding author.

## Supplementary Materials

The supporting information can be downloaded at https://www.mdpi.com/article/10.3390/ijms26167767/s1.

## Abbreviations

The following abbreviations are used in this manuscript:

ABGA	Anti-Basal Ganglia Antibody
ADHD	Attention Deficit Hyperactivity Disorder
AE	Autoimmune Encephalitis
ASD	Autism Spectrum Disorder
BBB	Blood–Brain Barrier
CD	Communication Disorder
CINs	Cholinergic Interneurons
CNS	Central Nervous System
CSF	Cerebrospinal Fluid
D2R	Anti-D2 Dopamine Receptor
DSM-5	Diagnostic and Statistical Manual of Mental Disorder-5
EEG	Electroencephalogram
GABHS	Group A-beta-hemolytic Streptococcus
GAS	Group A Streptococcus
GWAS	Genome-Wide Association Study
HLA	Human Leukocyte Antigen
HPA	Activation of the Hypothalamus–Pituitary–Adrenal
ID	Intellectual Disability
IFN-γ	Interferon-gamma
Ig	Immunoglobulin
M1	Microglia Phenotype Proinflammatory
M2	Microglia Phenotype Anti-Inflammatory
MIA	Maternal Immune Activation
MRI	Magnetic Resonance Imaging
NDDs	Neurodevelopmental Disorders
NMDAR	Anti-N-Methyl-D-Aspartate Receptor
NSE	Neuron-Specific Enolase
OCD	Obsessive–Compulsive Disorder
PANDAS	Pediatric Autoimmune Neuropsychiatric Disorders Associated With Group A β-hemolytic Streptococcus
PANS	Pediatric Acute-Onset Neuropsychiatric Syndrome
PD	Psychotic Disorder
PIDs	Primary Immunodeficiency Disorders
SAA	Serum Amyloid A
SCZ	Schizophrenia
SHH	Sonic Hedgehog Protein
SLD	Specific Learning Disorder
SNP	Single-Nucleotide Polymorphism
SS	Schizophrenia Spectrum
TD	Tic Disorder
TGF-β	Transforming Growth Factor-beta
TNF α	Tumor Necrosis Factor-alpha
TS	Tourette Syndrome
WES	Whole-Exome Study
WGS	Whole-Genome Sequencing Study
