# Supplementary material for: The Immune/Inflammatory Underpinnings of Neurodevelopmental Disorders and Pediatric Acute-Onset Neuropsychiatric Syndrome: A Scoping Review
Source: Int J Mol Sci. 2025 Aug 11;26(16):7767. doi: 10.3390/ijms26167767 (PMC12386443; doi:10.3390/ijms26167767)
Supplement: Supplementary file 1 [file ijms-26-07767-s001.zip › ijms-3726318-supplementary.pdf]

## SUPPLEMENTARY MATERIALS

**Supplementary Table S1:** PRISMA-ScR checklist ……………………………………………………………………………………………………………………………page 2-3

**Supplementary Table S2: Characteristics of included studies** …………………………………………………………………………………………………………….page 4-23

**This supplementary material has been provided by the authors to give readers additional information about their work.**

**Supplementary Table S1. Preferred Reporting Items for Systematic reviews and Meta-Analyses extension for Scoping Reviews (PRISMA-ScR) checklist.**

| SECTION | ITEM | PRISMA-ScR CHECKLIST ITEM | Location where item is reported |
|---|---|---|---|
| **TITLE** | | | |
| Title | 1 | Identify the report as a scoping review. | Title |
| **A** | | | |
| Structured summary | 2 | Provide a structured summary that includes (as applicable): background, objectives, eligibility criteria, sources of evidence, charting methods, results, and conclusions that relate to the review questions and objectives. | Abstract |
| **INTRODUCTION** | | | |
| Rationale | 3 | Describe the rationale for the review in the context of what is already known. Explain why the review questions/objectives lend themselves to a scoping review approach. | Introduction |
| Objectives | 4 | Provide an explicit statement of the questions and objectives being addressed with reference to their key elements (e.g., population or participants, concepts, and context) or other relevant key elements used to conceptualize the review questions and/or objectives. | Introduction |
| **METHODS** | | | |
| Protocol and registration | 5 | Indicate whether a review protocol exists; state if and where it can be accessed (e.g., a Web address); and if available, provide registration information, including the registration number. | Materials and Methods |
| Eligibility criteria | 6 | Specify characteristics of the sources of evidence used as eligibility criteria (e.g., years considered, language, and publication status), and provide a rationale. | Data Source and Search Strategy |
| Information sources* | 7 | Describe all information sources in the search (e.g., databases with dates of coverage and contact with authors to identify additional sources), as well as the date the most recent search was executed. | Materials and Methods |
| Search | 8 | Present the full electronic search strategy for at least 1 database, including any limits used, such that it could be repeated. | Materials and Methods |
| Selection of sources of evidence† | 9 | State the process for selecting sources of evidence (i.e., screening and eligibility) included in the scoping review. | Materials and Methods |
| Data charting process‡ | 10 | Describe the methods of charting data from the included sources of evidence (e.g., calibrated forms or forms that have been tested by the team before their use, and whether data charting was done independently or in duplicate) and any processes for obtaining and confirming data from investigators. | Materials and Methods |
| Data items | 11 | List and define all variables for which data were sought and any assumptions and simplifications made. | Materials and Methods |
| Critical appraisal of individual sources of evidence§ | 12 | If done, provide a rationale for conducting a critical appraisal of included sources of evidence; describe the methods used and how this information was used in any data synthesis (if appropriate). | Not Applicable |

| SECTION | ITEM | PRISMA-ScR CHECKLIST ITEM | Location where item is reported |
|---|---|---|---|
| Synthesis of results | 13 | Describe the methods of handling and summarizing the data that were charted. | Prisma, Figure 1 |
| **RESULTS** | | | |
| Selection of sources of evidence | 14 | Give numbers of sources of evidence screened, assessed for eligibility, and included in the review, with reasons for exclusions at each stage, ideally using a flow diagram. | Results |
| Characteristics of sources of evidence | 15 | For each source of evidence, present characteristics for which data were charted and provide the citations. | Results |
| Critical appraisal within sources of evidence | 16 | If done, present data on critical appraisal of included sources of evidence (see item 12). | Not Applicable |
| Results of individual sources of evidence | 17 | For each included source of evidence, present the relevant data that were charted that relate to the review questions and objectives. | Not Applicable |
| Synthesis of results | 18 | Summarize and/or present the charting results as they relate to the review questions and objectives. | Results |
| **DISCUSSION** | | | |
| Summary of evidence | 19 | Summarize the main results (including an overview of concepts, themes, and types of evidence available), link to the review questions and objectives, and consider the relevance to key groups. | Discussion |
| Limitations | 20 | Discuss the limitations of the scoping review process. | Limitations |
| Conclusions | 21 | Provide a general interpretation of the results with respect to the review questions and objectives, as well as potential implications and/or next steps. | Conclusion |
| **FUNDING** | | | |
| Funding | 22 | Describe sources of funding for the included sources of evidence, as well as sources of funding for the scoping review. Describe the role of the funders of the scoping review. | Funding |

**Supplementary Table S2. Characteristics of included studies.**

| Author | Year | | Title | Study Design | Sample Size | Presence of control group |
|---|---|---|---|---|---|---|
| Abdallah M.W. et al. | 2013 | | Neonatal Chemokine Levels and Risk of Autism Spectrum Disorders: Findings from a Danish Historic Birth Cohort Follow-up Study | Observational study(cohort, case–control, cross-sectional) | 1100 | ASD vs. frequency-matched controls |
| Abdallah M.W. et al. | 2012 | | Amniotic Fluid Chemokines and Autism Spectrum Disorders: An Exploratory Study Utilizing a Danish Historic Birth Cohort | Observational study (cohort, case–control, cross-sectional) | 1029 | ASD vs. healthy controls |
| Addabbo F. et al. | 2020 | | Anti-dopamine D2 Receptor Antibodies in Chronic Tic Disorders. | Observational study (cohort, case–control, cross-sectional) | 137 | no control group |
| Aguilar-Valles A. et al. | 2020 | | Maternal Immune Activation and the Development of Dopaminergic Neurotransmission of the Offspring: Relevance for Schizophrenia and Other Psychoses | Literature review | N.A. | N.A. |
| Ahmad SA. et al. | 2011 | | Seronegative limbic encephalitis: case report, literature review and proposed treatment algorithm. | Literature review | N.A. | N.A. |
| Al-Diwani A. et al. | 2019 | | The psychopathology of NMDAR-antibody encephalitis in adults: a systematic review and phenotypic analysis of individual patient data. | Literature review | N.A. | N.A. |

| | | | | | | |
|---|---|---|---|---|---|---|
| Al-Haddad B.J.S. et al. | 2019 | | Long-Term Risk of Neuropsychiatric Disease after Exposure to Infection in Utero. | Descriptive study | 1791520 | N.A. |
| Anand D. et al. | 2017 | | Attention-Deficit/Hyperactivity Disorder and Inflammation: What Does Current Knowledge Tell Us? A Systematic Review | Literature reviews | N.A. | N.A. |
| Bechter K. et al. | 2010 | | Cerebrospinal fluid analysis in affective and schizophrenic spectrum disorders: identification of subgroups with immune responses and blood–CSF barrier dysfunction | Observational study (cohort, case–control, cross-sectional) | 4163 | affective spectrum/schizophrenic spectrum vs. healthy controls |
| Benmakhlouf Y. et al. | 2020 | | Potential Cytokine Biomarkers in Intellectual Disability. Endocrine, Metabolic & Immune Disorders | Literature review | N.A. | N.A. |
| Benros ME. et al. | 2014 | | A nationwide study on the risk of autoimmune diseases in individuals with a personal or a family history of schizophrenia and related psychosis. | Observational study (cohort, case–control, cross-sectional) | 181692 | schizophrenia-like psychosis vs. autoimmune disease |
| Benros ME. et al. | 2011 | | Autoimmune diseases and severe infections as risk factors for schizophrenia: a 30-year population-based register study. | Descriptive study | 356757 | no control group |
| Benros ME. et al. | 2013 | | Autoimmune diseases and severe infections as risk factors for mood disorders: a nationwide study. | Descriptive study | 3.56 million | no control group |
| Berloffa S. et al. | 2023 | | Steroid treatment response to post SARS-CoV-2 PANS symptoms: Case series. | Descriptive study | 10 | no control group |
| Bjørklund G. et al. | 2016 | | Immune Dysfunction and Neuroinflammation in Autism Spectrum Disorder | Literature review | N.A. | N.A. |

| | | | | | | |
|---|---|---|---|---|---|---|
| Bos-Veneman N.G.P. et al. | 2011 | | Altered Immunoglobulin Profiles in Children with Tourette Syndrome | Observational study (cohort, case–control, cross-sectional) | 148 | Ts/tic vs. healthy controls |
| Brown A.S. et al. | 2015 | | Maternal Thyroid Autoantibody and Elevated Risk of Autism in a National Birth Cohort | Observational study (cohort, case–control, cross-sectional) | 1920 | ASD vs. without ASD or severe/profound intellectual disability |
| Brynge M. et al. | 2022 | | Maternal Infection during Pregnancy and Likelihood of Autism and Intellectual Disability in Children in Sweden: A Negative Control and Sibling Comparison Cohort Study. | Observational study (cohort, case–control, cross-sectional) | 549967 | maternal infection during pregnancy vs. negative control exposure |
| Buske-Kirschbaum A. et al. | 2013 | | Psychoendocrine and Psychoneuroimmunological Mechanisms in the Comorbidity of Atopic Eczema and ADHD. | Literature review | N.A. | N.A. |
| Cabrera B. et al. | 2019 | | Neuroanatomical features and its usefulness in classification of patients with PANDAS. | Observational study (cohort, case–control, cross-sectional) | 28 | patients with PANDAS vs. healthy subjects |
| Cainelli E. et al. | 2019 | | Neuropsychological and psychopathological profile of anti-NMDAR encephalitis: a possible pathophysiological model for pediatric neuropsychiatric disorders. | Observational study (cohort, case–control, cross-sectional) | 7 | no control group |
| CaiXiao JJ. et al. | 2018 | | Association between TNF-α-238G/A gene polymorphism and OCD susceptibility: a meta-analysis. | Literature review | N.A. | N.A. |
| Calaprice D. et al. | 2017 | | . A survey of pediatric acute-onset neuropsychiatric syndrome characteristics and course. | Descriptive study | 698 | no control group |

| | | | | | | |
|---|---|---|---|---|---|---|
| Chain JL. et al. | 2020 | | Autoantibody Biomarkers for Basal Ganglia Encephalitis in Sydenham Chorea and Pediatric Autoimmune Neuropsychiatric Disorder Associated With Streptococcal Infections. | Observational study (cohort, case–control, cross-sectional) | 96 | PANDAS and Sydenham Chorea vs. healthy controls |
| Chan A. et al. | 2022 | | Children With PANS May Manifest POTS. | Observational study (cohort, case–control, cross-sectional) | 204 | no control group |
| Chan A. et al. | 2020 | | Familial Clustering of Immune-Mediated Diseases in Children with Abrupt-Onset Obsessive Compulsive Disorder | Observational study (cohort, case–control, cross-sectional) | 365 | OCD and immune-mediated comorbidities in first-degree family members of patients and healthy controls |
| Chang K. et al. | 2015 | | Clinical evaluation of youth with pediatric acute-onset neuropsychiatric syndrome (PANS): recommendations from the 2013 PANS Consensus Conference. | Technical reports, theses, conference proceedings, book chapter | N.A. | N.A. |
| Chi S.H. et al. | 2023 | | Cytokine Levels Reflect Tic Symptoms More Prominently during Mild Phases. | Observational study (cohort, case–control, cross-sectional) | 66 | TIC disorder vs. healthy controls |
| Cieślik M. et al. | 2020 | | Maternal Immune Activation Induces Neuroinflammation and Cortical Synaptic Deficits in the Adolescent Rat Offspring | Observational study (cohort, case–control, cross-sectional) | 37 | LPS-treated dams vs. control |
| Congiu P. et al. | 2024 | | REM sleep atonia in patients with pediatric acute-onset neuropsychiatric syndrome: implications for pathophysiology. | Observational study (cohort, case–control, cross-sectional) | 113 | pediatric acute-onset neuropsychiatric syndrome vs. healthy controls |

| | | | | | | |
|---|---|---|---|---|---|---|
| Corona J.C. et al. | 2020 | | Role of Oxidative Stress and Neuroinflammation in Attention-Deficit/Hyperactivity Disorder | Literature review | N.A. | N.A. |
| Croen L. et al. | 2024 | | Inflammatory Conditions during Pregnancy and Risk of Autism and Other Neurodevelopmental Disorders. | Observational study (cohort, case–control, cross-sectional) | 2569 | ASD and DDs vs. healthy controls |
| Dale R.C. et al. | 2012 | | Antibodies to Surface Dopamine-2 Receptor in Autoimmune Movement and Psychiatric Disorders. | Observational study (cohort, case–control, cross-sectional) | 113 | Basal ganglia encephalitis vs. Sydenham's chorea vs. PANDAS vs. Ts |
| De Carvalho JF. et al. | 2020 | | Sjögren syndrome associated with obsessive-compulsive disorder | Literature review | N.A. | N.A. |
| De Giacomo A. et al. | 2021 | | B and T Immunoregulation: A New Insight of B Regulatory Lymphocytes in Autism Spectrum Disorder | Observational study (cohort, case–control, cross-sectional) | 42 | ASD vs. healthy controls |
| Donfrancesco R. et al. | 2020 | | Anti-Yo Antibodies in Children with ADHD: First Results about Serum Cytokines | Observational study (cohort, case–control, cross-sectional) | 94 | ADHD vs. healthy controls |
| Dunleavy C. et al | 2022 | | Inflammation in first-episode psychosis: the contribution of inflammatory biomarkers to the emergence of negative symptoms, a systematic review and meta-analysis. | Literature review | N.A. | N.A. |
| Dunn G. et al. | 2019 | | Neuroinflammation as a Risk Factor for Attention Deficit Hyperactivity Disorder | Literature review | N.A. | N.A. |
| Eaton WW. et al. | 2006 | | Association of schizophrenia and autoimmune diseases: linkage of Danish national registers | Comparative study | 200294 | schizophrenia patients vs. healthy controls |
| Elamin I. et al. | 2013 | | Immune Dysfunction in Tourette Syndrome. | Literature review | N.A. | N.A. |

| | | | | | | |
|---|---|---|---|---|---|---|
| Endres D. et al. | 2022 | | Immunological causes of obsessive-compulsive disorder: is it time for the concept of an "autoimmune OCD" subtype? | Literature review | N.A. | N.A. |
| Endres D. et al. | 2015 | | Immunological findings in psychotic syndromes: a tertiary care hospital's CSF sample of 180 patients. | Descriptive study | 180 | N.A. |
| Fasmer O.B. et al. | 2011 | | Adult attention deficit hyperactivity disorder Is Associated with Asthma. | Observational study (cohort, case–control, cross-sectional) | 1313 | ADHD vs. healthy control |
| Foroughipour M. et al. | 2012 | | Frequency of obsessive-compulsive disorder in patients with multiple sclerosis: a cross-sectional study. | Observational study (cohort, case–control, cross-sectional) | 112 | no control group |
| Frankovich J. et al. | 2015 | | Multidisciplinary clinic dedicated to treating youth with pediatric acute-onset neuropsychiatric syndrome: presenting characteristics of the first 47 consecutive patients. | Descriptive study | 47 | N.A. |
| Frick L. et al. | 2016 | | Microglial dysregulation in OCD, Tourette syndrome, and PANDAS. | Literature review | N.A. | N.A. |
| Frick L. R. et al. | 2018 | | Differential binding of antibodies in PANDAS patients to cholinergic interneurons in the striatum. | Randomized controlled trial (RCT) | 35 | PANDAS vs. healthy control |
| Gagliano A. et al. | 2021 | | Artificial neural networks analysis of polysomnographic and clinical features in pediatric acute-onset neuropsychiatric syndrome (PANS): from sleep alteration to .. | Descriptive study | 23 | N.A. |
| Gallego JA. et al. | 2018 | | Cytokines in cerebrospinal fluid of patients with schizophrenia spectrum disorders: new data and an updated meta-analysis. | Literature review | N.A. | N.A. |

| | | | | | | |
|---|---|---|---|---|---|---|
| Gaughan T. et al. | 2016 | | Rapid Eye Movement Sleep Abnormalities in Children with Pediatric Acute-Onset Neuropsychiatric Syndrome (PANS). | Descriptive study | 15 | N.A. |
| Geller H. et al. | 2021 | | 6.34 Comparison of Primary Humoral Immunodeficiencies in Autism Spectrum Disorder (ASD) and Other Pediatric-Onset Psychiatric Disorders | Technical reports, theses, conference proceedings, book chapter | N.A. | N.A. |
| Gerentes M. et al. | 2019 | | Obsessive-compulsive disorder: autoimmunity and neuroinflammation. | Literature review | N.A. | N.A. |
| Giana G.et al. | 2015 | | Detection of Auto-Antibodies to DAT in the serum: Interactions with DAT Genotype and psycho-stimulant therapy for ADHD. | Descriptive study | 38 | ADHD vs. healthy controls |
| Giedd JN. et al. | 2000 | | MRI assessment of children with obsessive-compulsive disorder or tics associated with streptococcal infection. | Descriptive study | 116 | OCD/TIC vs. healthy controls |
| Girgis RR. et al. | 2018 | | A randomized, double-blind, placebo-controlled clinical trial of tocilizumab, an interleukin-6 receptor antibody, for residual symptoms in schizophrenia. | Randomized controlled trial (RCT) | 36 | TCZ vs. placebo |
| Giri YR. et al. | 2021 | | Anti-N-methyl-D-aspartate receptor (NMDAR) encephalitis in children and adolescents: a systematic review and quantitative analysis of reported cases. | Literature review | N.A. | N.A. |
| Gładysz D. et al. | 2018 | | Immune Abnormalities in Autism Spectrum Disorder—Could They Hold Promise for Causative Treatment? | Literature review | N.A. | N.A. |

| | | | | | | |
|---|---|---|---|---|---|---|
| Goldsmith DR. et al. | 2016 | | A meta-analysis of blood cytokine network alterations in psychiatric patients: comparisons between schizophrenia, bipolar disorder and depression. | Literature review | N.A. | N.A. |
| Graham A.M. et al. | 2018 | | Maternal Systemic Interleukin-6 During Pregnancy Is Associated with Newborn Amygdala Phenotypes and Subsequent Behavior at 2-Years-of-Age. | Observational study (cohort, case–control, cross-sectional) | 86 | no control group |
| Graus F. et al. | 2016 | | A clinical approach to diagnosis of autoimmune encephalitis. | Literature review | N.A. | N.A. |
| Gray SM. et al. | 2012 | | Systematic review of proinflammatory cytokines in obsessive-compulsive disorder. | Literature review | N.A. | N.A. |
| Gromark C. et al. | 2019 | | Establishing a pediatric acute-onset neuropsychiatric syndrome clinic: Baseline clinical features of the pediatric acute-onset neuropsychiatric syndrome cohort at Karolinska Institutet | Observational study (cohort, case–control, cross-sectional) | 45 | no control group |
| Gromark C. et al. | 2022 | | A two-to-five year follow-up of a pediatric acute-onset neuropsychiatric syndrome cohort. | Observational study (cohort, case–control, cross-sectional) | 34 | no control group |
| Han V.X. et al. | 2021 | | Maternal Immune Activation and Neuroinflammation in Human Neurodevelopmental Disorders. | Literature review | N.A. | N.A. |
| Hardy D. | 2022 | | Autoimmune encephalitis in children | Literature review | N.A. | N.A. |
| Harris J. | 2006 | | Intellectual Disability: Understanding Its Development, Causes, Classification, Evaluation, and Treatment. | Technical reports, theses, conference proceedings, book chapter | N.A. | N.A. |

| | | | | | | |
|---|---|---|---|---|---|---|
| Hegvik T.A. et al | 2018 | | Associations between attention-deficit/hyperactivity disorder and autoimmune diseases are modified by sex: a population-based cross-sectional study | Observational study (cohort, case–control, cross-sectional) | 2500118 | ADHD vs. healthy controls |
| Hegvik T.A. et al. | 2022 | | Familial Co-Aggregation of attention-deficit/hyperactivity disorder and Autoimmune Diseases: A Cohort Study based on Swedish population-wide registers | Observational study (cohort, case–control, cross-sectional) | 5178225 | ADHD vs. healthy controls |
| Heuer L.S. et al. | 2019 | | An Exploratory Examination of Neonatal Cytokines and Chemokines as Predictors of Autism Risk: The Early Markers for Autism Study. | Observational study (cohort, case–control, cross-sectional) | 888 | ASD/ID vs. healthy controls |
| Hu C. et al. | 2022 | | Microglia: Synaptic Modulator in Autism Spectrum Disorder | Literature review | N.A. | N.A. |
| Huang X.et al. | 2021 | | Peripheral Inflammation and Blood–Brain Barrier Disruption: Effects and Mechanisms | Literature review | N.A. | N.A. |
| Hughes H.K. et al. | 2023 | | Innate Immune Dysfunction and Neuroinflammation in Autism Spectrum Disorder (ASD). | Literature review | N.A. | N.A. |
| Instanes J.T. et al. | 2017 | | Attention-deficit/hyperactivity disorder in Offspring of Mothers with Inflammatory and Immune system Diseases. | Observational study (cohort, case–control, cross-sectional) | 2322657 | ADHD vs. healthy controls |
| Jellinger K.A. | 2019 | | Neuropathology and Pathogenesis of Extrapyramidal Movement Disorders: A Critical Update. II. Hyperkinetic Disorders | Literature review | N.A. | N.A. |
| Johnson M. et al. | 2019 | | Paediatric acute-onset neuropsychiatric syndrome in children and adolescents: an observational cohort study | Observational study (cohort, case–control, cross-sectional) | 415 | no control group |

| | | | | | | |
|---|---|---|---|---|---|---|
| Jones K.L. et al. | 2017 | | Autism with Intellectual Disability Is Associated with Increased Levels of Maternal Cytokines and Chemokines during Gestation | Descriptive study | 1031 | ASD/DDs vs. healthy controls |
| Kalinowski A. et al. | 2023 | | Evaluation of C4 gene copy number in Pediatric Acute Neuropsychiatric Syndrome | Descriptive study | 303 | PANS vs. healthy controls |
| Kawikova I. et al. | 2007 | | Decreased Numbers of Regulatory T Cells Suggest Impaired Immune Tolerance in Children with Tourette Syndrome: A Preliminary Study | Descriptive study | 46 | TC/OCD/TIC+OCD vs. healthy controls |
| Kayser MS. et al. | 2016 | | Anti-NMDA receptor encephalitis, autoimmunity, and psychosis. | Literature review | N.A. | N.A. |
| Keski-Rahkonen A. et al. | 2023 | | Avoidant-Restrictive Food Intake Disorder and Autism: Epidemiology, Etiology, Complications, Treatment, and Outcome | Literature review | N.A. | N.A. |
| Keszler G. et al. | 2014 | | Association of the Tumor Necrosis Factor -308 A/G Promoter Polymorphism with Tourette Syndrome | Descriptive study | 1004 | ADHD/OCD/TS/Tic vs. healthy controls |
| Khandaker GM. et al. | 2014 | | Association of serum interleukin 6 and C-reactive protein in childhood with depression and psychosis in young adult life: a population-based longitudinal study | Observational study (cohort, case–control, cross-sectional) | 4415 | no control group |
| Krakowiak P. et al. | 2017 | | Neonatal Cytokine Profiles Associated with Autism Spectrum Disorder | Observational study (cohort, case–control, cross-sectional) | N.A. | N.A. |

| | | | | | | |
|---|---|---|---|---|---|---|
| Kumar A. et al. | 2015 | | Evaluation of Basal Ganglia and Thalamic Inflammation in Children With Pediatric Autoimmune Neuropsychiatric Disorders Associated With Streptococcal Infection and Tourette Syndrome: A Positron Emission Tomographic (PET) Study Using 11C-[R]-PK11195 | Descriptive study | 44 | PANDAS/TS vs. healthy controls |
| Lampiasi N. et al. | 2023 | | Inflammation and the Potential Implication of Macrophage-Microglia Polarization in Human ASD: An Overview | Literature review | N.A. | N.A. |
| Leckman J.F. et al. | 2005 | | Increased Serum Levels of Interleukin-12 and Tumor Necrosis Factor-Alpha in Tourette's Syndrome | Randomized controlled trial (RCT) | 77 | TS vs. healthy controls |
| Leffa D. et al. | 2019 | | A Review on the Role of Inflammation in attention-deficit/hyperactivity disorder | Literature review | N.A. | N.A. |
| Leonardi L. et al. | 2024 | | Pediatric acute-onset neuropsychiatric syndrome (PANS) and pediatric autoimmune neuropsychiatric disorders associated with streptococcal infections (PANDAS): immunological features underpinning controversial entities. | Literature review | N.A. | N.A. |
| Li D.J. et al. | 2022 | | Associations between Allergic and Autoimmune Diseases with autism spectrum disorder and attention-deficit/hyperactivity disorder within Families: a population-based cohort study | Observational study (cohort, case–control, cross-sectional) | 1386260 | no control group |
| Li Y. et al. | 2022 | | Profiles of Proinflammatory Cytokines and T Cells in Patients With Tourette Syndrome: A Meta-Analysis | Literature review | N.A. | N.A. |

| | | | | | | |
|---|---|---|---|---|---|---|
| Liao T.C. et al. | 2016 | | Comorbidity of Atopic Disorders with autism spectrum disorder and attention deficit/hyperactivity disorder | Observational study (cohort, case–control, cross-sectional) | 774524 | ASD/ADHD vs. healthy controls |
| Lim M. et al. | 2023 | | PANDAS/PANS in the COVID-19 age: Autoimmunity and Epstein–Barr virus reactivation as trigger agents? | Literature review | N.A. | N.A. |
| Lin Y.T. et al. | 2016 | | Associations between Allergic Diseases and attention deficit hyperactivity/oppositional defiant disorders in Children | Descriptive study | 2896 | N.A. |
| Ma M. et al. | 2024 | | Development of Autoimmune Diseases Among Children With Pediatric Acute-Onset Neuropsychiatric Syndrome. | Observational study (cohort, case–control, cross-sectional) | 193 | no control group |
| Madra M. et al. | 2020 | | Gastrointestinal Issues and Autism Spectrum Disorder. | Literature review | N.A. | N.A. |
| Martino D. et al. | 2015 | | The Role of Immune Mechanisms in Tourette Syndrome. | Literature review | N.A. | N.A. |
| Mataix-Cols D. et al. | 2018 | | A Total-Population Multigenerational Family Clustering Study of Autoimmune Diseases in Obsessive–Compulsive Disorder and Tourette's/Chronic Tic Disorders. | Observational study (cohort, case–control, cross-sectional) | 37361 | OCD vs. healthy controls |
| McKeon A. et al. | 2020 | | Autoimmune psychosis. | Technical reports, theses, conference proceedings, book chapter | N.A. | N.A. |
| Mekori-Domachevs.ky E. et al. | 2017 | | Elevated Proinflammatory Markers in 22q11.2 Deletion Syndrome Are Associated with Psychosis and Cognitive Deficits. | Descriptive study | 79 | Down Syndrome vs. healthy controls |

| | | | | | | |
|---|---|---|---|---|---|---|
| Melamed I. et al. | 2024 | | IVIG response in pediatric acute-onset neuropsychiatric syndrome correlates with reduction in pro-inflammatory monocytes and neuropsychiatric measures. | Descriptive study | 10 | N.A. |
| Metcalf SA. et al. | 2017 | | Serum C-reactive protein in adolescence and risk of schizophrenia in adulthood: a prospective birth cohort study. | Observational study (cohort, case–control, cross-sectional) | 6362 | no control group |
| Miller B.J. et al. | 2011 | | Meta-Analysis of Cytokine Alterations in Schizophrenia: Clinical Status and Antipsychotic Effects. | Technical reports, theses, conference proceedings, book chapter | N.A | N.A. |
| Murphy TK. et al. | 2013 | | Tonsillectomies and adenoidectomies do not prevent the onset of pediatric autoimmune neuropsychiatric disorder associated with group A streptococcus. | Randomized controlled trial (RCT) | 108 | PANS vs. healthy controls |
| Murphy TK. et al. | 2012 | | Clinical factors associated with pediatric autoimmune neuropsychiatric disorders associated with streptococcal infections. | Descriptive study | 109 | N.A. |
| Najjar S. et al. | 2018 | | A clinical approach to new-onset psychosis associated with immune dysregulation: the concept of autoimmune psychosis. | Theoretical or conceptual paper | N.A. | N.A. |
| Nayak U. et al. | 2025 | | Impact of neuroinflammation on brain glutamate and dopamine signalling in schizophrenia: an update. | Literature review | N.A. | N.A. |

| | | | | | | |
|---|---|---|---|---|---|---|
| Nielsen T.C. et al. | 2022 | | Association of Maternal Autoimmune Disease and Early Childhood Infections with Offspring Autism Spectrum Disorder: A Population-Based Cohort Study. | Observational study (cohort, case–control, cross-sectional) | 55353 | children exposed to maternal autoimmune disease vs. unexposed children |
| O'Loughlin E. et al. | 2017 | | Acute in Utero Exposure to Lipopolysaccharide Induces Inflammation in the Pre- and Postnatal Brain and Alters the Glial Cytoarchitecture in the Developing Amygdala | Observational study (cohort, case–control, cross-sectional) | N.A. | no control group |
| Oades R.D. et al. | 2010 | | Attention-Deficit Hyperactivity Disorder (ADHD) and Glial Integrity: S100B, Cytokines and Kynurenine Metabolism—Effects of Medication | Observational study (cohort, case–control, cross-sectional) | 63 | ADHD vs. healthy controls |
| Orlovs.ka S. et al. | 2017 | | Association of streptococcal throat infection with mental disorders: testing key aspects of the PANDAS hypothesis in a nationwide study. | Observational study (cohort, case–control, cross-sectional) | 349982 | no control group |
| Oskvig D.B. et al. | 2012 | | Maternal Immune Activation by LPS Selectively Alters Specific Gene Expression Profiles of Interneuron Migration and Oxidative Stress in the Fetus without Triggering a fetal immune Response | Descriptive study | 34 | maternal immune activation vs. healthy controls |
| Pavone P. et al. | 2020 | | Severe psychotic symptoms in youth with PANS/PANDAS: case-series. | Observational study (cohort, case–control, cross-sectional) | 8 | no control group |
| Pearlman DM. et al. | 2014 | | Anti-basal ganglia antibodies in primary obsessive-compulsive disorder: systematic review and meta-analysis. | Literature review | N.A. | N.A. |

| | | | | | | |
|---|---|---|---|---|---|---|
| Pohlman D. | 2018 | | PANDAS Network: PN 2018 State of Our Children SURVEY | Technical reports, theses, conference proceedings, book chapter | N.A. | N.A. |
| Pollak T. et al. | 2018 | | The blood–brain barrier in psychosis. | Literature review | N.A. | N.A. |
| Pollak T. et al. | 2020 | | Autoimmune psychosis: an international consensus on an approach to the diagnosis and management of psychosis of suspected autoimmune origin. | Theoretical or conceptual paper | N.A. | N.A. |
| Rezaeinejad M. et al. | 2023 | | The Association between Maternal Infection and Intellectual Disability in Children: A Systematic Review and Meta-Analysis. | Literature review | N.A. | N.A. |
| Robbins TW. et al. | 2019 | | Obsessive-compulsive disorder: puzzles and prospects | Literature review | N.A. | N.A. |
| Robertson M.M. et al. | 2017 | | Gilles de la Tourette Syndrome. | Literature review | N.A. | N.A. |
| Rodriguez J.I. et al. | 2011 | | Evidence of Microglial Activation in Autism and Its Possible Role in Brain Underconnectivity | Literature review | N.A. | N.A. |
| Rodriguez N. et al. | 2019 | | Human-leukocyte antigen class II genes in early-onset obsessive-compulsive disorder. | Descriptive study | 144 | N.A. |
| Rosenberg J.B. et al. | 2024 | | Maternal Inflammation during Pregnancy Is Associated with Risk of ADHD in Children at Age 10. | Observational study (cohort, case–control, cross-sectional) | 604 | ADHD vs. healthy controls |
| Rudolph M.D. et al. | 2018 | | Maternal IL-6 during Pregnancy Can Be Estimated from Newborn Brain Connectivity and Predicts Future Working Memory in Offspring | Descriptive study | 84 | N.A. |
| Ruscio AM. et al. | 2010 | | The epidemiology of obsessive-compulsive disorder in the National Comorbidity Survey Replication | Descriptive study | 2073 | N.A. |

| | | | | | | |
|---|---|---|---|---|---|---|
| Saini T. et al. | 2025 | | New-onset OCD and juvenile enthesitis-related arthritis after COVID-19 (Three Cases). | Observational study (cohort, case–control, cross-sectional) | 3 | no control group |
| Santoro JD. et al. | 2018 | | Continued Presence of Period Limb Movements During REM Sleep in Patients With Chronic Static Pediatric Acute-Onset Neuropsychiatric Syndrome (PANS). | Descriptive study | 9 | N.A. |
| Segman R.H. et al. | 2002 | | Preferential Transmission of interleukin-1 Receptor Antagonist Alleles in attention deficit hyperactivity disorder. | Descriptive study | 85 | N.A. |
| Silverman M. et al. | 2019 | | Psychotic symptoms in youth with Pediatric Acute-onset Neuropsychiatric Syndrome (PANS) may reflect syndrome severity and heterogeneity. | Descriptive study | 143 | N.A. |
| Snider LA. et al. | 2004 | | PANDAS: current status and directions for research. | Literature review | N.A. | N.A. |
| Stagi S. et al. | 2014 | | Evaluation of autoimmune phenomena in patients with pediatric autoimmune neuropsychiatric disorders associated with streptococcal infections (PANDAS) | Literature review | N.A. | N.A. |
| Subeh GK. et al. | 2021 | | Anti-N-methyl-D-aspartate receptor encephalitis: a detailed review of the different psychiatric presentations and red flags to look for in suspected cases. | Literature review | N.A. | N.A. |

| | | | | | | |
|---|---|---|---|---|---|---|
| Swedo S.E. et al. | 1998 | | Pediatric Autoimmune Neuropsychiatric Disorders Associated with Streptococcal Infections: Clinical Description of the First 50 Cases. Obsessive-Compulsive Disorder and Tourette's Syndrome | Descriptive study | 50 | N.A. |
| Swedo SE. et al. | 2012 | | From research subgroup to clinical syndrome: modifying the PANDAS criteria to describe PANS (Pediatric Acute-Onset Neuropsychiatric Syndrome). | Theoretical or conceptual paper | N.A. | N.A. |
| Tamouza R. et al. | 2021 | | Understanding the genetic contribution of the human leukocyte antigen system to common major psychiatric disorders in a world pandemic context. | Literature review | N.A. | N.A. |
| Tao Y. et al. | 2022 | | Changes of Cytokines in Children With Tic Disorder. | Randomized controlled trial (RCT) | 2274 | Tic vs. healthy controls |
| Than U.T.T. et al. | 2023 | | Inflammatory Mediators Drive Neuroinflammation in Autism Spectrum Disorder and Cerebral Palsy | Observational study (cohort, case–control, cross-sectional) | 62 | no control group |
| Thomas F. et al. | 2025 | | IL-17 in serum and cerebrospinal fluid of pediatric patients with acute neuropsychiatric disorders: Implications for PANDAS and PANS | Observational study (cohort, case–control, cross-sectional) | 58 | no control group |
| Toto M. et al. | 2015 | | Antibasal Ganglia Antibodies and Antistreptolysin O in Noncomorbid ADHD | Observational study (cohort, case–control, cross-sectional) | 40 | ADHD vs. healthy controls |
| Trépanier MO. Et al. | 2016 | | Postmortem evidence of cerebral inflammation in schizophrenia: a systematic review. | Literature review | N.A. | N.A. |

| | | | | | | |
|---|---|---|---|---|---|---|
| Trifiletti R. et al. | 2022 | | Identification of Ultra-Rare Genetic Variants in Pediatric Acute Onset Neuropsychiatric Syndrome (PANS) by Exome and Whole Genome Sequencing | Observational study (cohort, case–control, cross-sectional) | 646 | PANS vs. healthy controls |
| Tsetsos F. et al. | 2021 | | Synaptic Processes and Immune-Related Pathways Implicated in Tourette Syndrome. | Observational study (cohort, case–control, cross-sectional) | 7682 | no control group |
| Tylee D.S. et al. | 2018 | | Genetic Correlations among Psychiatric and Immune-related Phenotypes Based on Genome-wide Association Data. | Literature review | N.A. | N.A. |
| Upthegrove R. et al. | 2014 | | Cytokine function in medication-naive first-episode psychosis: a systematic review and meta-analysis. | Literature review | N.A. | N.A. |
| Usui N. et al. | 2023 | | Neuroinflammation and Oxidative Stress in the Pathogenesis of Autism Spectrum Disorder | Literature review | N.A. | N.A. |
| van Kesteren CF. et al. | 2017 | | Immune involvement in the pathogenesis of schizophrenia: a meta-analysis on postmortem brain studies. | Literature review | N.A. | N.A. |
| Vargas D.L. et al. | 2005 | | Neuroglial Activation and Neuroinflammation in the Brain of Patients with Autism | Observational study (cohort, case–control, cross-sectional) | 17 | ASD vs. healthy controls |
| Vázquez-González D. et al. | 2023 | | A Potential Role for Neuroinflammation in ADHD | Literature review | N.A. | N.A. |
| Villarreal V.R. et al. | 2024 | | Risk of Autoimmune Disease in Research-Identified Cases of Autism Spectrum Disorder: A Longitudinal, Population-Based Birth Cohort Study. | Observational study (cohort, case–control, cross-sectional) | 3042 | ASD vs. healthy controls |

| | | | | | | |
|---|---|---|---|---|---|---|
| Voineagu I. et al. | 2011 | | Transcriptomic Analysis of Autistic Brain Reveals Convergent Molecular Pathology. | Observational study (cohort, case–control, cross-sectional) | 36 | ASD vs. healthy controls |
| Wang LY. et al. | 2019 | | Systemic autoimmune diseases are associated with an increased risk of obsessive-compulsive disorder: a nationwide population-based cohort study. | Observational study (cohort, case–control, cross-sectional) | 378990 | SADs or without SADs |
| Weidinger E. et al. | 2014 | | Impaired Activation of the Innate Immune Response to Bacterial Challenge in Tourette Syndrome. | Observational study (cohort, case–control, cross-sectional) | 64 | Ts vs. healthy controls |
| Williams KA. et al. | 2016 | | Randomized, controlled trial of intravenous immunoglobulin for pediatric autoimmune neuropsychiatric disorders associated with streptococcal infections. | Randomized controlled trial (RCT) | 35 | PANDAS vs. healthy controls |
| Xiong Y. et al. | 2023 | | Microglia and Astrocytes Underlie Neuroinflammation and Synaptic Susceptibility in Autism Spectrum Disorder | Literature review | N.A. | N.A. |
| Xu J. et al. | 2021 | | Antibodies from children with PANDAS bind specifically to striatal cholinergic interneurons and alter their activity. | Observational study (cohort, case–control, cross-sectional) | 50 | PANDAS vs. healthy controls |
| Xu J. et al. | 2022 | | Elevated antibody binding to striatal cholinergic interneurons in patients with pediatric acute-onset neuropsychiatric syndrome | Observational study (cohort, case–control, cross-sectional) | 50 | PANS vs. healthy controls |
| Yolken RH. et al. | 2008 | | Are some cases of psychosis caused by microbial agents? A review of the evidence. | Literature review | N.A. | N.A. |

| | | | | | | |
|---|---|---|---|---|---|---|
| Zebrack JE. et al. | 2025 | | Neurological Soft Signs at Presentation in Patients With Pediatric Acute-Onset Neuropsychiatric Syndrome. | Observational study (cohort, case–control, cross-sectional) | 119 | no control group |
| Zerbo O. et al. | 2015 | | Immune Mediated Conditions in Autism Spectrum Disorders. | Observational study (cohort, case–control, cross-sectional) | 33390 | ASD vs. healthy controls |
| Zhang F. et al. | 2025 | | Blood–Brain Barrier Disruption in Schizophrenia: Insights, Mechanisms, and Future Directions | Literature review | N.A. | N.A. |
| Zhang T. et al. | 2023 | | Prenatal and early childhood infections and subsequent risk of obsessive-compulsive disorder and tic disorders: a nationwide, sibling-controlled study. | Observational study (cohort, case–control, cross-sectional) | 281089 | individuals exposed to any prenatal maternal infection vs. siblings to control for familial confounding |
| Zheng J. et al. | 2020 | | Association of Pediatric Acute-Onset Neuropsychiatric Syndrome With Microstructural Differences in Brain Regions Detected via Diffusion-Weighted Magnetic Resonance Imaging. | Observational study (cohort, case–control, cross-sectional) | 98 | PANS vs. healthy controls |

**Legend:** ASD = autism spectrum disorder; ADHD = attention deficit/hyperactivity disorder; ID = intellectual disability; DDs = developmental disorders; N.A. = not applicable; OCD = obsessive–compulsive disorder; TCZ= Tocilizumab; PANDAS= Pediatric Autoimmune Neuropsychiatric Disorders Associated with Streptococcal Infections; PANS= Pediatric Acute-Onset Neuropsychiatric Syndrome; TS= Tourette syndrome
